# Revisiting the development of cerebellar inhibitory interneurons in the light of single-cell genetic analyses

**DOI:** 10.1007/s00418-023-02251-z

**Published:** 2023-11-08

**Authors:** Karl Schilling

**Affiliations:** Anatomisches Institut – Anatomie und Zellbiologie, Rheinische Friedrich-Wilhelms-Universität Bonn, Nussallee 10, 53115 Bonn, Germany

**Keywords:** Cerebellum, Development, Interneuron, Inhibitory neurons, Neuronal migration, scRNA

## Abstract

The present review aims to provide a short update of our understanding of the inhibitory interneurons of the cerebellum. While these cells constitute but a minority of all cerebellar neurons, their functional significance is increasingly being recognized. For one, inhibitory interneurons of the cerebellar cortex are now known to constitute a clearly more diverse group than their traditional grouping as stellate, basket, and Golgi cells suggests, and this diversity is now substantiated by single-cell genetic data. The past decade or so has also provided important information about interneurons in cerebellar nuclei. Significantly, developmental studies have revealed that the specification and formation of cerebellar inhibitory interneurons fundamentally differ from, say, the cortical interneurons, and define a mode of diversification critically dependent on spatiotemporally patterned external signals. Last, but not least, in the past years, dysfunction of cerebellar inhibitory interneurons could also be linked with clinically defined deficits. I hope that this review, however fragmentary, may stimulate interest and help focus research towards understanding the cerebellum.

## Introduction

The cerebellum has fascinated neuroscientists and clinicians alike from early on and has served again and again as a paradigm to study neural structure, function, and development.

The cerebellar cortex is often used as a textbook example to introduce fundamental morphological and functional aspects of neuronal circuits, but also of developmental processes underpinning the formation of the nervous system. Its attraction as a model may be traced to its apparently simple cellular composition and highly stereotyped synaptic connectivity, as beautifully described and presaged early on by Ramon y Cajal (1909). Since then, a wealth of studies have turned the cerebellum into a Rosetta stone describing mechanistic principles and molecular correlates of and prerequisites for the formation of complex neuronal circuits and their glial complement. These studies also revealed the molecular and mechanistic bases of clinically significant neurological deficits related to cerebellar pathology (for reviews, see e.g. Manto et al. 2021).

Analysis of cerebellar development and function greatly profited from the availability of a set of murine mutants with prominent and often strikingly specific cerebellar phenotypes (Caviness and Rakic 1978). Eventually, this allowed the direct genetic targeting of the two most prominent cerebellar cell populations, i.e. Purkinje cells (Oberdick et al. 1988, 1990) and granule cells (Ben-Arie et al. 1997; Jensen et al. 2002; Gliem et al. 2006), and their in-depth molecular characterization, arguably best documented in our by now highly detailed understanding of the origins and molecular make-up of medulloblastoma and its relation to the lineages of glutamatergic neurons (i.e. granule cells, unipolar bush cells, and nuclear rhombic lip-derived neurons; e.g., Hendrikse et al. 2022; Williamson et al. 2022; Vladoiu et al. 2019 to cite only a few more recent papers; for Purkinje cells, see Chen et al. 2022).

**Fig. 1:**
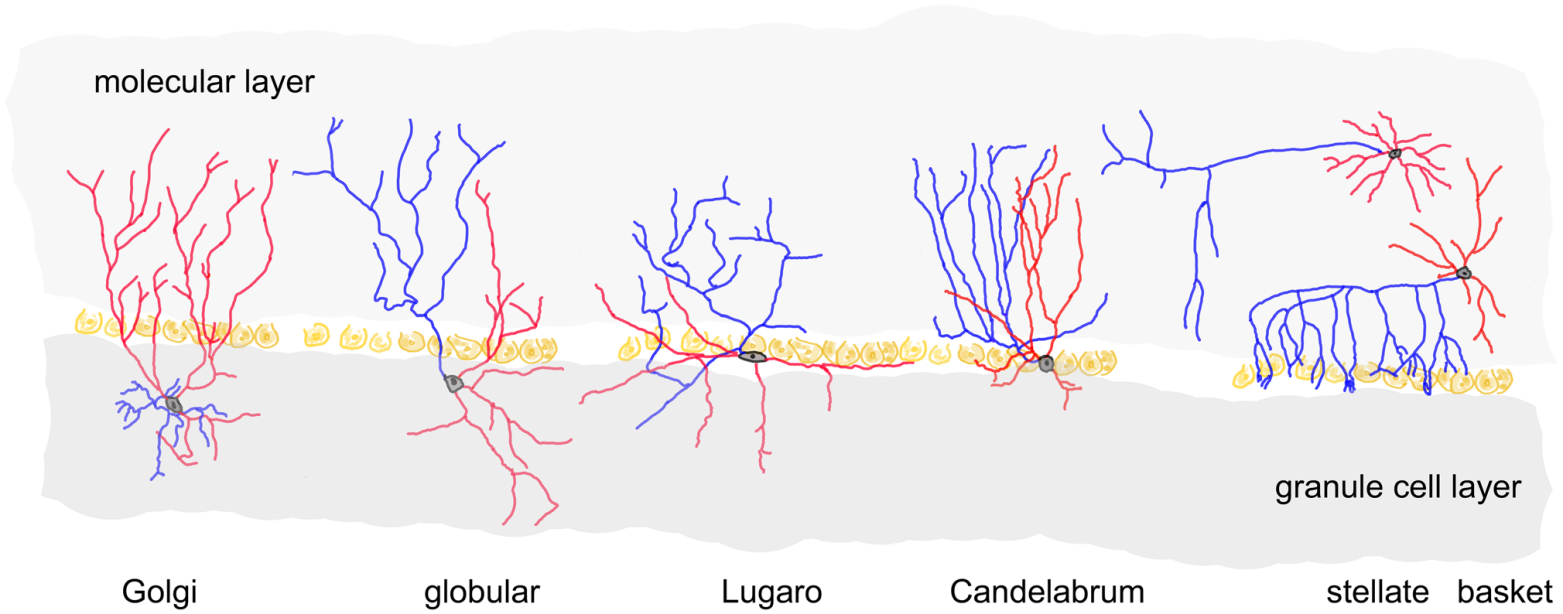
A schematic view of the locations of the somata (black/gray), the distribution of the dendrites (red), and the axonal spread (blue) of inhibitory interneurons of the cerebellar cortex. This sketch was inspired by camera lucida drawings by Ramon y Cajal (1909), Palay and Chan-Palay (1974), and Laine and Axelrad (1994, 1996, 2002). Note that this scheme does not reflect the intricate detail of the neurites of these cells. Purkinje cell somata are indicated in ochre

In striking contrast, our understanding of and our experimental access to cerebellar inhibitory interneurons and GABAergic and/or glycinergic neurons in the cerebellar nuclei is still quite limited. One stumbling block not yet overcome is that their precursors are so far not selectively accessible for molecular manipulation (for an example to the contrary described for adult molecular layer interneurons, see Amat et al. 2017). In the present review, I try to provide a current perspective of the development, classification, and molecular characterization of cerebellar inhibitory interneurons (Fig. 1), including those of the cerebellar nuclei. I build on previous reviews of this topic (e.g., Leto et al. 2016; Hibi et al. 2017; Kano et al. 2018; Schilling 2019; Haldipur et al. 2022), and I focus primarily on data that became available since this topic was reviewed here some 15 years ago (Schilling et al. 2008). I also try to incorporate data extracted from publicly available single-cell gene expression (scRNA) datasets, primarily those accompanying the publications of Carter et al. (2018), Vladoiu et al. (2019), Kozareva et al. (2021), and Khouri-Farah et al. (2022). To illustrate gene expression patterns, representative images are taken from the Allen Brain Atlas (Lein et al. 2007; see https://developingmouse.brain-map.org/). Lastly, this review is limited essentially to data obtained with mice as a model system, unless otherwise indicated.

## Defining precursors of inhibitory interneurons: the establishment of inhibitory and excitatory lineages in the nascent cerebellum

The cerebellum is derived from the dorsal (alar) plate of rhombomere 1 (r1; Wassef 2022; note that r1 is used here to encompass r0 and r1 as defined by Puelles and associates; cf. Aroca and Puelles 2005). Early on, two distinctive neurogenic regions may be recognized in the cerebellar anlage, the dorsally located rhombic lip and the more ventrally located ventricular epithelium. Whereas the former gives rise to excitatory (glutamatergic) neurons, the latter is the source of inhibitory (GABAergic and/or glycinergic)^1^ neurons, cerebellar astroglia, and a minor subset of cerebellar oligodendrocytes (for details, see Consalez et al. 2021; Hibi and Shimizu 2012; Leto et al. 2012; Cerrato et al. 2018; Hashimoto et al. 2016).

The identification of the transcription factors Ptf1a (Hoshino et al. 2005; Pascual et al. 2007) and Atoh1 (Ben-Arie et al. 1997) as critical for the development of cerebellar inhibitory and excitatory neuronal lineages, respectively, constituted significant technical breakthroughs in the quest to understand the genetic and mechanistic basis of cerebellar development. Among the questions that these discoveries made accessible, two appear particularly intriguing: First, how do Ptf1a- and Atoh1-defined cells arise, and second, how do these lineages, and especially the lineage(s) giving rise to inhibitory interneurons, further diversify?

In the nascent cerebellar anlage, Atoh1 is a convenient marker for the rhombic lip, from which excitatory neurons of the cerebellar nuclei, granule cells precursors, and unipolar brush cells arise. The extensive and intriguing advances in our understanding of these cell lineages and their clinical significance will not be further considered here. The interested reader may be referred to recent publications by Consalez et al. (2021), Kebschull et al. (2023), and Hendrikse and associates (Hendrikse et al. 2022).

Conversely, in the nascent cerebellum, Ptf1a labels much of the ventricular neuroepithelium, the source of all but one subset of cerebellar inhibitory neurons (for this one exception see below, “Diversification of cerebellar inhibitory cells (projection neurons vs. interneurons)”). As recently shown by Zhang et al. (2021), both Ptf1a-defined precursors of inhibitory and Atoh1-defined precursors of excitatory cerebellar cells are derived from a common pool of Sox2-expressing “naïve” neuroepithelial cells. Cell fate decisions that eventually lead to the establishment of these lineages (and presumably the astrocytic lineage as well) are based on Notch-Rbpj signaling. Sox2-positive neuroepithelial cells express high levels of Notch-Rbpj activity. They may develop into cells with an excitatory fate in which Notch-Rbpj activity is low, or into cells destined to become inhibitory neurons, characterized by intermediate Notch-Rbpj activity levels. Notch activity in the cerebellar anlage is regulated by its trans-acting ligand, Dll1, as well as by its cis-inhibiting ligand, Dll3. Thus, differential expression of Notch1 and its ligands Dll1 and Dll3 bring about cell fate decision in the nascent cerebellar anlage and also constitute one of the earliest indicators of prospective cell fate. Consistent with this model is the earlier observation that ablation of Rbpj in precursors of molecular layer inhibitory interneurons greatly reduces their numbers (Komine et al. 2011). Note that in this study, Rbpj signaling was disturbed exclusively in astroglial cells and in the molecular layer interneuron lineage(s). Consequently, it does not allow us to infer or exclude an effect of Notch signaling on the generation of other populations of inhibitory interneurons, or on that of Purkinje cells or nucleo-olivary projection neurons.

As observed in various systems, notably the spinal cord and retina, Notch-Rbpj signaling is also closely integrated with the expression of several neurogenic genes, including Ascl1 (Shi et al. 2012; Nelson et al. 2009), Bhlhe22 (Skaggs et al. 2011), Neurog2 (Henke et al. 2009), Gsx1 (Tzatzalos et al. 2012), and Prdm13 (Hanotel et al. 2014), which are all also expressed in the ventricular epithelium of the cerebellar anlage. As Ptf1a directly interacts with Rbpj (Chang et al. 2013), it may be seen as a key hub in this integration.

## Patterns in the cerebellar anlage: some cues from the spinal cord

The dorsoventral subdivision of the early cerebella anlage revealed by Atoh1 and Ptf1a suggests that understanding its further maturation and cellular diversification may profit from a comparison with the developing spinal cord, in which dorsoventral patterning has been studied extensively. Indeed, several genes indicative of the dorsoventral differentiation of the alar (dorsal) plate of the developing spinal cord (e.g., Atoh1, Ptf1a, Neurog1, Neurog2, Ascl1, Lmx1a, Lhx1, Lhx5, Pax2, Dbx1; Butler and Bronner 2015; Wilson and Maden 2005; Sagner and Briscoe 2019; Delile et al. 2019) are also expressed in the cerebellar anlage, where their spatial expression pattern mirrors that seen in the spinal cord (for details, see below). Another example of a parallel pattern of gene expression is provided by Bhlhe22 (Ross et al. 2012; Skaggs et al. 2011). In the spinal cord, this gene is first selectively expressed in early-born interneurons in the dorsal precursor domain dI6 and the ventral domains V1 and V2; as development proceeds, it is also expressed in two additional sets of dorsal interneurons referred to as dIL^A^ and dIL^B^, which reach their positions in the superficial laminae rather late (Liu et al. 2007). Similarly, in the cerebellar anlage, Bhlhe22 is expressed early on at the level corresponding to the spinal domain dl6 (which is referred to as pc4; see below), and, with a protracted time course, also in derivatives of both excitatory and inhibitory lineages that derive from regions other than pc4.

Yet there are also some noteworthy differences in gene expression patters in the developing spinal cord and the early cerebellar anlage. Notably, Olig2, which is expressed strictly ventrally in the developing spinal cord, where it is involved in motor neuron specification (Delile et al. 2019), is expressed in the dorsally derived cerebellar anlage. The same is true for Sox14, which is expressed only ventrally in the spinal cord anlage (Hargrave et al. 2000; Katsuyama et al. 2022), but transiently labels prospective nucleo-olivary projection neurons in the developing cerebellum (Prekop et al. 2018). Lastly, it should be recalled that Hox genes are not expressed in the territory (r1) from which the cerebellum arises (Alexander et al. 2009). Indeed, if expression of the only Hox gene expressed in r2, Hox2a, is abrogated, this rhombomere contributes to the development of a (laterally) enlarged cerebellum (Gavalas et al. 1997).

**Fig. 2:**
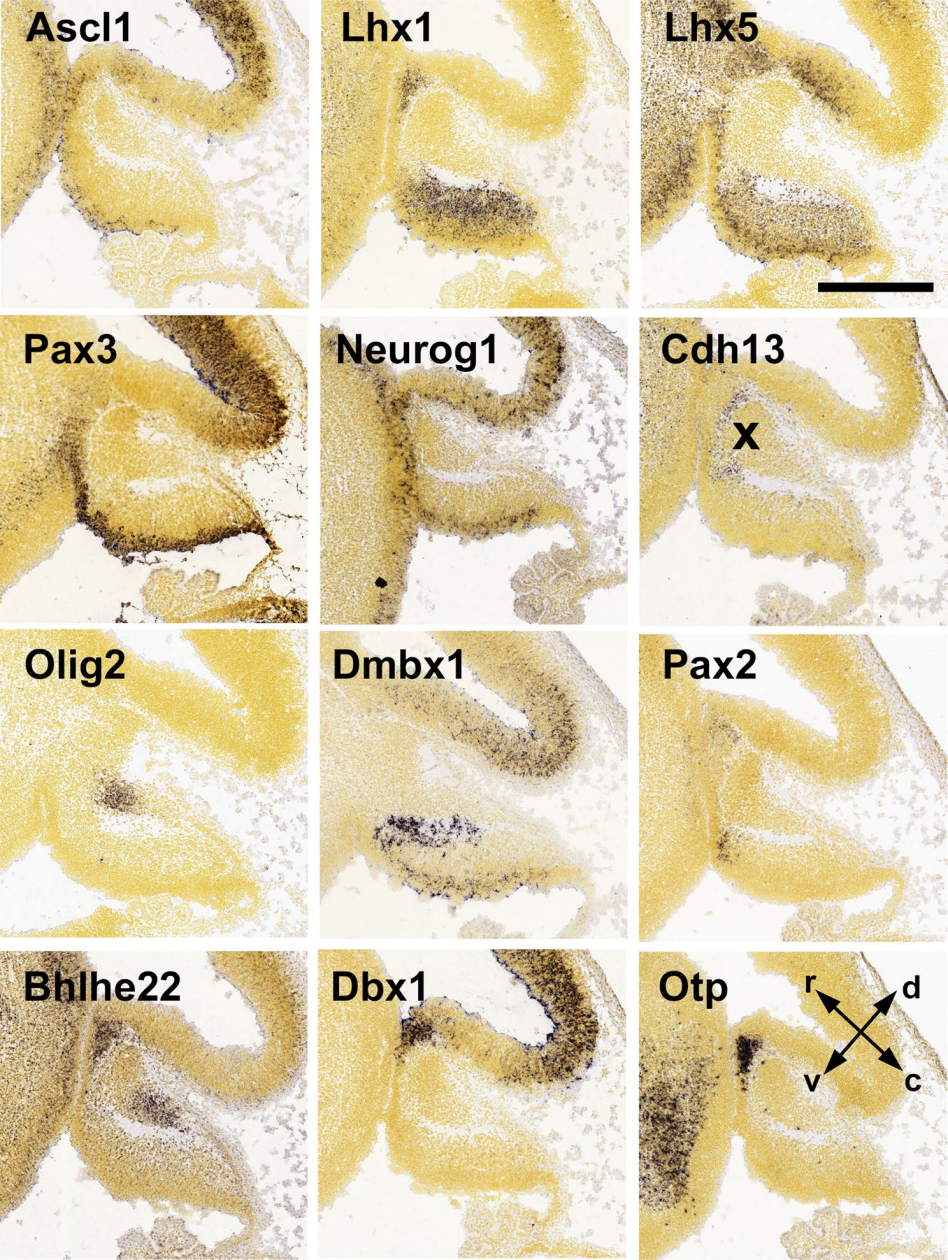
In situ hybridization for molecular markers of precursors of inhibitory neurons in the cerebellar anlage at embryonic day 13.5 (E13.5). Ascl1, Lhx1, Lhx5, Pax3, and Neurog1 are early markers expressed throughout the ventricular zone, with a preferential expression either in the ventricular epithelium (Ascl1, Pax3) or in cells that have just left the epithelial layer (Lhx1, Lhx5, Neurog1). Note also the Lhx1/5 difference, which is reflected, during later development, in a slightly preferential expression of Lhx1 in prospective Purkinje neurons and of Lhx5 in prospective inhibitory interneurons. Cadherin 13 (Cdh13) expression can be seen in two regions slightly rostral and either dorsal or ventral to the anlage of the deep nuclei (the latter is marked by an “x” in this panel). Note that this label closely resembles that seen for Pax2 at this age. At E13.5, Olig2 is no longer expressed in Purkinje cell precursors, but expression is still visible in a set of prospective excitatory deep nuclear neurons (see main text, and also Fig. 3 for details). Dmbx1, a marker for prospective nucleo-olivary projection neurons, can be seen expressed in the ventricular zone and at the ventral margin of the nuclear anlage in which these cells integrate. Bhlhe22, Dbx1, and Otp mark the ventricular zone in the rostral-most region of the cerebellar anlage, referred to as pc4. Bhlhe22 expression can also be seen in cells of the nuclear anlage not related to the ventricular cells expressing this marker. Arrows (in panel “Otp”) indicate rostral (r), caudal (c), dorsal (d), and ventral (v); scale bar = 0.5 mm. All images were taken from the Allen Brain Atlas (Lein et al. 2007)

All these genes, those with expression patterns reminiscent of the developing spinal cord and those that show a divergent pattern, may be used to further subdivide the nascent cerebellar anlage, and notably its ventricular epithelium. Canonically, the early cerebellar ventricular epithelium is subdivided into three regions, referred to, from rostral to caudal, as pc4, pc3, and pc2 [(p)c1 would be the rhombic lip], based primarily on the differential expression of Ascl1, Neurog1, and Neurog2 (Chizhikov et al. 2006; Zordan et al. 2008). These regions have in turn have been associated with the developmental origin of specific populations of cerebellar inhibitory projection neurons and inhibitory interneurons, respectively. For a detailed discussion and illustration of the subdivision of the cerebellar ventricular zone, see Zordan et al. (2008). Figure 2 summarizes the regional expression of some key genes in the cerebellar anlage at embryonic day 13.5 (E13.5). As shall be discussed below, the expression patterns of these genes change dynamically as development goes on, so these images rather reflect a snapshot.

## Diversification of cerebellar inhibitory cells (projection neurons vs. interneurons)

Before discussing the genetic diversification of cerebellar inhibitory neurons, a brief reminder of the classification of these cells may be appropriate. The mammalian cerebellum comprises at least three major classes of projection neurons utilizing either GABA or glycine as transmitters: Purkinje cells, nucleo-olivary projection neurons, and the glycinergic nuclear neurons projecting to the brainstem, originally described by Bagnall et al. in 2009 (see also Kebschull et al. 2023).

Purkinje cells, which provide the sole output of the cerebellar cortex, and nucleo-olivary projection neurons, connecting the cerebellar nuclei to the inferior olive, are both GABAergic. They have been extensively characterized in the adult, and they are readily distinguished from GABAergic/glycinergic interneurons by morphological, functional, and molecular criteria. Both are derivatives of Ptf1a-positive precursors of the ventricular epithelium. As will be detailed below, early markers of these cells are Olig2 and Dmbx1, respectively. In contrast, the glycinergic nuclear projection neurons identified by Bagnall et al. (2009) stand out due to their developmental origin from Atoh1-positive precursors originating in the rhombic lip (Kebschull et al. 2020). Based on their overall pattern of gene expression, these cells closely resemble glutamatergic, rhombic-lip-derived nuclear projection neurons of the cerebellum. Thus, these cells are not developmentally related to other cerebellar GABAergic or glycinergic neurons, and their transmitter phenotype is rather due to a neurotransmitter switch (Kebschull et al. 2020; for a review on transmitter switching, see Spitzer 2015).

Formally, the GABAergic neurons that connect the fastigial nuclei on both sides of the cerebellum (Gomez-Gonzalez and Martinez-Torres 2021) also qualify as projection neurons. The developmental history of these cells is still obscure. Curiously, at least a subset of these cells also express Pax2 (Gomez-Gonzalez and Martinez-Torres 2021). This is of particular interest, since Pax2 is generally taken as one of the markers (or rather, the marker) that distinguishes the (early) lineages of cerebellar GABAergic and/or glycinergic interneurons from GABAergic projection neurons.

In fact, a common and defining feature of cerebellar inhibitory interneurons is that they are all derived from precursors expressing Pax2 at about the time they go through their last mitosis. While some cerebellar inhibitory interneurons continue to express Pax2 into adulthood, most of them do not (Maricich and Herrup 1999; Weisheit et al. 2006; see also supplementary material to Kebschull et al. 2020). The classification of Pax2-derived inhibitory interneurons has been reviewed previously (Schilling et al. 2008), and in “From Pax2 cells to specific types of cerebellar interneurons,” this classification will be revisited in the light of recent single-cell gene expression data.

## Some notes on key genes that pattern the cerebellar ventricular epithelium or may be used as markers of distinct inhibitory lineages

How then do the genes mentioned above relate to the establishment of projection and/or interneuronal lineages? The seminal work of Hoshino et al. (2005; see also Pascual et al. 2007) established that **Ptf1a** is indispensable for the development of all cerebellar inhibitory neurons. **Prdm13,** which is a direct downstream target of Ptf1a (Bessodes et al. 2017), has been variably implicated in the generation of Purkinje neurons (Coolen et al. 2022) or Purkinje neurons as well as Pax2-positive precursors of inhibitory interneurons (Whittaker et al. 2021). As of this writing, it cannot be concluded whether these divergent results reflect differences in species (humans, mice, and zebrafish were used for analyses) or in the mutations analyzed, or even differences in the experimental focus of these studies. Both studies concur, though, that Prdm13 is needed, in mammals, for the proper differentiation of Purkinje neurons. Also, whether Prdm13 also affects the development of nucleo-olivary projection neurons has not (yet) been formally probed. Interrogation of recent single-cell gene expression studies (Khouri-Farah et al. 2022; Vladoiu et al. 2019; Carter et al. 2018) reveals that Prdm13 is expressed not only in Olig2-positive precursors of Purkinje cells and Pax2-positive cells, but also in Dmbx1-positive cells of the cerebellar anlage. Thus, Prdm13, like Ptf1a, is coexpressed with all markers that eventually allow us to define all inhibitory lineages arising from the ventricular epithelium. The data by Khouri-Farah et al. (2022) indicate that between embryonic days 10 and 17, Prdm13 is expressed in some 11.1% of cells also positive for Dmbx1. For comparison, it is found in about 22.3% of all Olig2-positive precursors of Purkinje cells, and in about 2.8% of all Pax2-positive cells in this data set. Consistent numbers are obtained with the data from Vladoiu et al. (2019; age range, embryonic day 10 to postnatal day 14; 8.5%, 27.6%, and 6.1% for Dmbx1-, Olig2-, and Pax2-positive cells, respectively). These numbers must be taken with a grain of salt, given the known variability in single-cell gene expression studies, which are all the more pronounced the fewer cells that are available for analysis. Still, the differences in the percentages of Prdm13-positive cells in the various lineages is fully consistent with the developmental timing of the expression of the markers used to identify these lineages, and the fact that Prdm13, like Ptf1a, is expressed only in early ventricular epithelial cells of the cerebellum (Coolen et al. 2022, and further references given there).

In the spinal cord, Prdm13 also ensures that genes specifying ventral neural tube development, including Olig2, are not expressed dorsally (Mona et al. 2017). Yet, as its coexpression with Olig2 in the cerebellar anlage documents, the function of Prdm13 in the (dorsal) spinal cord and the cerebellar anlage must differ at least in part.

**Alscl1** is expressed early on in precursors of inhibitory neurons of the cerebellar nuclei and cortex (Kim et al. 2008; Grimaldi et al. 2009; Sudarov et al. 2011). In Ascl1-deficient mice, generation of Pax2-defined inhibitory interneurons is severely impaired, whereas “no major effect” on Purkinje cells was observed in this genotype (Grimaldi et al. 2009). Interestingly, the effect of Ascl1-ablation was more pronounced for late-born inhibitory interneurons of the upper molecular layer, and rather mild for the earlier-born inhibitory interneurons resident in the granule cell layer (Sudarov et al. 2011). The effects on inhibitory neurons of the cerebellar nuclei were so far not tested. Considering the temporal sequence in which Purkinje neurons and inhibitory interneurons are generated (become postmitotic; cf. Leto et al. 2009), one may generalize that these observations suggest that Ascl1 is the more important the longer precursors originating from the cerebellar ventricular epithelium continue to proliferate. Moreover, Ascl1 also balances the development of inhibitory interneurons and astroglial cells, as reported by Grimaldi et al. (2009). Their data show that over-expression of Ascl1 in the ventricular epithelium of the cerebellar anlage increases the numbers of Pax2-positive precursors of inhibitory interneurons and reduces the numbers of cells of the astroglial lineage. Ablation of Ascl1 expression in the cerebellar ventricular zone had the converse effect.

Beyond the critical effect of Ascl1 on the ratio of Purkinje cells and Pax2-expressing cells, the balancing of these cell types is also tuned by the basic helix-loop-helix transcription factor, **Olig2** (Seto et al. 2014b). In the early cerebellar anlage, say up to embryonic day 14, Olig2 is expressed in two spatially and molecularly well separated sets of cells, i.e., precursors of Purkinje neurons and a group of nuclear neurons (for further details, see below). Later it is also expressed by precursors of oligodendrocytes.

Precursors of cerebellar Purkinje cells are strongly positive for Olig2 up to embryonic day 12.5, yet Olig2 is not required for Purkinje cell formation per se; rather, its ablation, when combined with ablation of Olig1, which may compensate Olig2 function, results in reduced Purkinje cell numbers and a concomitant increase in **Gsx1**-positive neuroepithelial cells, from which Pax2-positive interneuronal precursors arise (Seto et al. 2014b). These authors also remark that they “could not detect any cells that expressed both transcription factors [i.e., Olig2 and Gsx1] simultaneously,” and conclude that “even at the earliest stage of cerebellar GABAergic neuron production, there exist two populations of GABAergic progenitors, PIPs [Pax2-positive interneuron progenitors] and PCPs [Purkinje cell progenitors]”—implying that Gsx1 and Olig2 are not at the very root of the splitting of these two populations. This conclusion may have to be reconsidered given that single-cell gene expression data reveal coexpression of Olig2 and Gsx1, if only in low numbers of cells. Thus, the data of Khouri-Farah et al. (2022) indicate that between e10 and e17, Olig2 is expressed in 4.9% of all Gsx1-positive cells, and Gsx1 in 2.9% of all Olig2-positive cells. Consistent numbers may be obtained by probing the data of Vladoiu et al. (2019). Taking into account that at these ages, Olig2 is also expressed in a few early-forming oligodendrocytes, and the abovementioned Olig2-positive cerebellar nuclear cells, the fraction of Olig2-expressing cells within the neuroepithelium also expressing Gsx1 may well be larger than this percentage indicates. The study by Seto et al. (2014b) further documents that the Olig2-positive domain is progressively exhausted as cerebellar development proceeds. Concomitantly, the Gsx1-defined territory expands, reminiscent of the distinct temporal patterns with which Purkinje cells and Pax2-positive cells emerge.

In slight contrast to Seto et al. (2014b), Ju et al. (2016) reported that deletion of Olig2 alone (i.e., without simultaneous ablation of Olig1) is sufficient to reduce the numbers of Purkinje cells, without having an appreciable effect on numbers of Pax2-positive cells. The basis of these discrepancies is not known. It may reflect differences in the knock-out mouse models and their background strains used, or even slight differences in developmental stages analyzed (although these were nominally identical). Be that as it may, both studies concur in showing that manipulation of Olig2 results in a shift in the Purkinje cell/inhibitory interneuron ratio.

The balancing between Olig2-positive precursors of Purkinje cells and Gsx1-expressing precursors of Pax2-defined inhibitory interneurons was recently linked to the dynamic changes in SMAD/BMP-signaling during cerebellar development (Ma et al. 2020). These authors observed that high levels of BMP/SMAD activity repress Gsx1 transcription and thus inhibit the formation of cells of the inhibitory interneuron lineage. As development proceeds, SMAD activity declines. Subsequently, expression of Gsx1 is initiated, first in the ventral and later also in the dorsal ventricular epithelium, and allows the formation of Pax2-positive interneuronal precursors. Intriguingly, these observations suggest a mechanistic link between cellular diversification within the cerebellar anlage and its early induction following expression of Fgf8 within the isthmic organizer. To wit, Fgf8 is not only one of the first molecular markers that makes it possible to distinguish midbrain and future cerebellar territory; it is, when expressed ectopically, also capable of inducing the formation of cerebellar cells/tissue (e.g., Hidalgo-Sanchez et al. 2022, and Wassef 2022 for a recent reviews). Conversely, following ablation of Fgf8, neither a cerebellum nor a proper midbrain develops (Sato and Joyner 2009; Hörnblad et al. 2021). Significantly, Fgf8 and BMPs have been recognized as antagonistic developmental regulators in several systems, including the cerebellar anlage (e.g., Neubüser et al. 1997; Hörnblad et al. 2021; Wall and Hogan 1995; Basson et al. 2008; for further references, see also Siebel and Lendahl 2017). Finally, it should be recalled that the mitotic expansion of the ventricular zone is influenced by signals originating in the (dorsal) roof plate and fourth ventricular choroid plexus (for a recent review, see Chizhikov 2021).

Together, these data clearly indicate that the cerebellar ventricular zone is a more dynamic and plastic region than its subdivision in the abovementioned zones might suggest. Indeed, these zones should rather be perceived as snapshots of a dynamically changing pattern of gene expression taken at well-defined developmental time points.

Finally, a few words about the Olig2-positive cell group in the nascent cerebellar nuclei seem warranted. As indicated, Olig2 is expressed in these cells up to about embryonic day 14 (Wizeman et al. 2019; Ju et al. 2016; Seto et al. 2014a, b; see also the Allen Brain Atlas). The origin of these cells has so far not been identified, nor is it known whether they are related to Olig2-positive precursors of Purkinje cells. Ju et al. (2016) speculated that these nuclear postmitotic Olig2-positive cells might be derived from Olig2-positive cells in the ventricular epithelium. Conversely, the tight spatial integration of nuclear Olig2-positive cells with other groups of rhombic lip-derived nuclear cells suggests that they might derive from the rhombic lip. Thus, in the E13.5 cerebellar anlage, there is spatial overlap between Olig2-positive nuclear cells and rhombic-lip-derived nuclear cells (Wizeman et al. 2019). In contrast, Dmbx1-positive prospective nucleo-olivary projection neurons, which are unambiguously derived from the ventricular epithelium (Prekop et al. 2018; Kebschull et al. 2023), are well separated from Olig2-positive nuclear cells in the cerebellar anlage (compare panels “Olig2” and “Dmbx1” in Fig. 2).

**Fig. 3:**
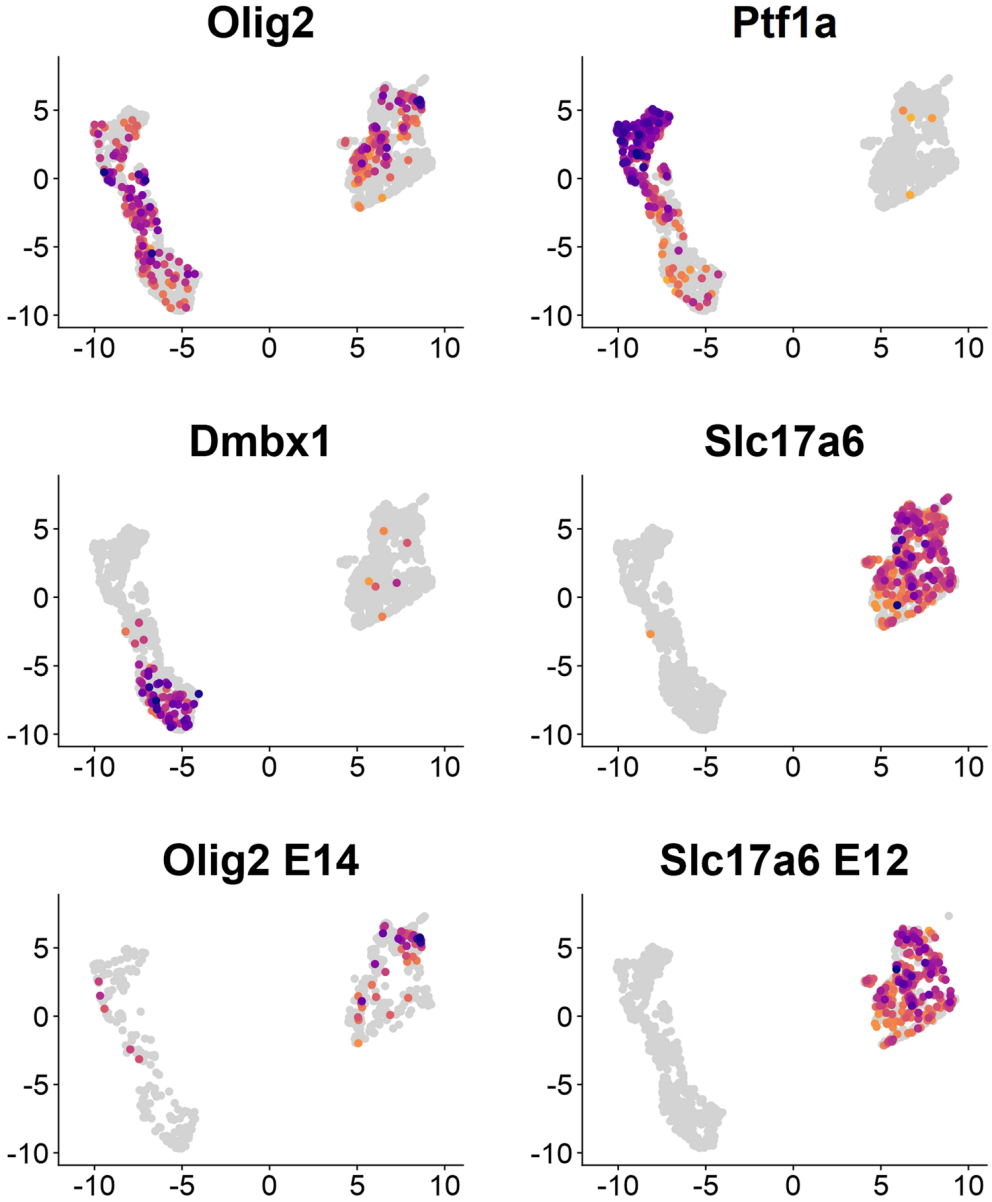
Clustering of Olig2-positive cells from embryonic murine cerebella obtained at E12 and E14 (upper four panels) or selectively at E12 or E14 (bottom panels). Olig2-positive cells are found in two well-separated clusters, one of which is also labeled by established markers for cerebellar inhibitory lineages (of which Ptf1a and Dmbx1 are shown), and the other is labeled by the neuronal vesicular glutamate transporter, Slc17a6. The bottom panels show that Olig2 expression in inhibitory precursors is much reduced at E14, and that the excitatory differentiation marker Slc17a6 is already expressed at E12, consistent with an early maturation of this group of Olig2-expressing cells. Data from Vladoiu et al. (2019) were processed following a standard workflow in R (R Core Team 2023) using package Seurat 4.3.1 (Hao et al. 2021). Cells obtained from 12- and 14-day-old embryonic tissues were used for clustering, and clusters positive for Olig2 were isolated. Data were visualized using package scCustomize (Marsh 2023). Darker colors indicate stronger expression

Interrogation of publicly available single-cell gene expression data from the developing cerebellum now allows us to approach this issue. As the data of Vladoiu et al. (2019) reveal (Fig. 3), Olig2 cells from E12 and E14 cerebella, i.e., at the ages that bracket the main expression period described for ventricular and nuclear Olig2-positive cells (Seto et al. 2014a; Wizeman et al. 2019), segregate into two well-separated groups of clusters. These are characterized by coexpression of either a set of established markers of the cerebellar inhibitory lineages, including Ptf1a, Prdm13, Tfap2a, Tfap2b, Kirrel2, Dmbx1, and Neurog1, or with markers for (excitatory) rhombic-lip derived nuclear neurons, including Lhx9 and the vesicular glutamate transporter, Slc17a6. The latter group is also characterized by expression of Meis2, Nrp1, and the neurofilament genes Nefl and Nefm. Together, this suggests that these cells are, even at E12, at an advanced stage of differentiation, which agrees with the observation of Ju et al. (2016) that nuclear Olig2-positive cells are postmitotic. Consistently, expression of the classical markers for the external granule cell layer, Atoh1 and Barhl1, could be seen only in a subset of nuclear Olig2-positive cells. While these data do not provide formal proof that nuclear Olig2-positive cells are derived from the rhombic lip, they strongly suggest so; in any case, they clearly indicate that these cells develop into excitatory cells. Lastly, neither of these two early Olig2-positive cell populations expresses markers indicative of the oligodendrocyte lineage. Consistent data can be obtained with the scRNA data of Carter et al. (2018).

Whether and how the recently identified function of Olig3 as yet another regulator of the balanced generation of Purkinje cells and inhibitory interneuron fits in this scheme remains to be answered. As shown by Lowenstein et al. (2021), ablation of Olig3 in (presumptive) Purkinje cell precursors (identified by their location in the pc2 domain) re-specified these cells to the Pax2 lineage.

**Dmbx1,** which marks precursors and maturing nucleo-olivary projection neurons**,** codes for a paired-like homeodomain protein originally described to be expressed in the developing diencephalon and midbrain (Ohtoshi et al. 2002; Ohtoshi and Behringer 2004; Takahashi and Holland 2004; and further references there). Based on in situ hybridization data available in the Allen Brain Atlas (Lein et al. 2007) and the scRNA data of Kebschull et al. (2020), its expression is maintained well beyond P14. At first glance, this appears at conflict with developmental scRNA studies, in which no cells positive for Dmbx1 could be observed in cerebella obtained from animals older than, say, P7 (Carter et al. 2018; Vladoiu et al. 2019). However, beyond that age, granule cells progressively outnumber all other cerebellar cells, and the granule cell to nuclear cell ratio is well beyond 500 (numbers from Heckroth 1994; Surchev et al. 2007). Thus, it comes as no surprise that in samples that at best comprise a few thousand cells per age group analyzed (Carter et al. 2018; Vladoiu et al. 2019), cells projecting to the inferior olive, which account for only a minor fraction of all nuclear cells, are not represented.

Gene ablation studies (Ohtoshi and Behringer 2004) showed that Dmbx1 is needed for the generation of a full complement of nucleo-olivary projection neurons, but not of these cells per se. Thus, mice in which one of the two Dmbx1 genes was interrupted by insertion of lacZ developed four times the number of beta-galactosidase-positive nucleo-olivary projecting neurons found in mice carrying two null genes (one again tagged by lacZ).

**Bhlhe22** (formerly Bhlhb5), the Antp-type homeobox gene **Dbx1**, and the paired-type homeobox gene, **Otp** (Simeone et al. 1994), all mark the pc4 region of the cerebellar anlage (Fig. 2). The developmental fate of cells from this region and their contribution to the mature cerebellum, or even to extracerebellar structures, is not really understood.

Similar to Olig2, its related bHLH gene Bhlhe22 (Ross et al. 2012) is expressed in the ventricular zone of the nascent cerebellar anlage and in a subset of nuclear neurons. And like Olig2, Blhle22-expressing cells cluster in well-separated sets characterized by expression of markers for ventricular-zone- and rhombic lip-derived cells, respectively. Interrogation of scRNA data from Vladoiu et al. (2019) confirms the impression conveyed by the spatial expression patterns of Dbx1, Olig2, and also Bhlhe22 that these genes are indeed coexpressed in individual cells. In fetuses aged 12 and 14 days, coexpression can be seen in ~ 54–60% of the cerebellar cells expressing any one of these genes. Coexpression with Pax2 could be ascertained in 57/149 Otp-positive cells, in 50/189 Dbx1-positive cells, and in 31/415 Bhlhe22-positive cells in samples spanning E12–E18 (data from Vladoiu et al. 2019). While samples of E12 also comprise brain stem cells (Vladoiu et al. 2019), in which Pax2 may be expressed, the observation of Bhlhe22/Pax2 double-positive cells, if few, supports the overall interpretation that Otp/Dbx1/Bhlhe22-positive cells from pc4 contribute to cerebellar inhibitory interneurons. It does not exclude the possibility that they also contribute to extracerebellar structures. Consistent coexpression patterns may be derived from Khouri-Farah et al. (2022; see also the website associated with this publication), which cover the age range E10–E17, with brainstem data from E10 and E12.

Unfortunately, neither Bhlh22, nor Dbx1, nor Otp have so far been able to be utilized to directly track the developmental fate of the pc4 cells that express these genes at E13.5. As indicated, Bhlhe22 is also expressed, at later stages, in the external granule cell layer and its derivatives (Ramirez et al. 2022; see also the Allen Brain Atlas). Dbx1 and Otp are not expressed beyond E14 in the cerebellar anlage (Allen Brain Atlas and data above). Intriguingly, though, in E13.5 cerebella, two groups of cells positive for Cdh13 can be seen next to or even overlapping with those positive for Dbx1, Otp, Bhlhe22, and Pax2 (see Fig. 2). Analysis of the scRNA data of Vladoiu et al. (2019) and Carter et al. (2018) confirms cellular coexpression of Cdh13 with Pax2, Otp, and Bhlhe22. The data of Carter et al. (2018) also suggest coexpression of Cdh13 with Dbx1. Despite this minor discrepancy, these data consistently suggest that the Pax2/Otp/Bhlhe22- and possibly Dbx1-defined cell population of pc4 contributes to cerebellar inhibitory interneurons, and specifically to those in the granule cell layer and deep nuclei, which are the only populations of cerebellar inhibitory interneurons expressing Cdh13 in the adult (Schilling and Oberdick 2010).

A common feature of Olig2, Dmbx1, Dbx1, and also of Pax2, which marks the inhibitory interneuron lineage (more about this in the next section), is that they are all expressed around the last mitosis within the cell lineages they are specific for (Ju et al. 2016; Wong et al. 2015; Pierani et al. 2001; Weisheit et al. 2006). As just discussed, these genes are critical for the adequate formation and numerical matching of Purkinje cells, nucleo-olivary projecting neurons, and inhibitory cerebellar interneurons, and may be used as lineage markers. Yet this should not distract from the fact, revealed more recently by scRNA expression studies, that in early development, sizable fractions of cells coexpress at least two of these markers, as is well documented by data reported in independent single-cell gene expression studies (Carter et al. 2018; Vladoiu et al. 2019; Khouri-Farah et al. 2022). The actual degree of coexpression revealed by these data is quite variable. This may reflect differences in the developmental stages analyzed, but also methodological variability. Still, the data accord to show that cell-type/lineage-specific expression of these genes is preceded by a phase of coexpression. Whether the transition to lineage-specific expression indicates differential regulation by external (cell extrinsic) signals or a signaling gradient, or whether this involves the mutual repression of the genes we use as lineage markers, or possibly both mechanisms, remains to be studied.

## From Pax2 cells to specific types of cerebellar interneurons

The paired-box gene Pax2 is the earliest and a unique marker for the precursors of all cerebellar inhibitory interneurons. Before reviewing the scarce information we have about mechanisms bringing about the diversification of Pax2-defined precursors, it seems sensible to briefly recall how cerebellar inhibitory interneurons are classified in the adult, and how this classification has gained from recent single-cell (scRNA) analyses. The functional significance of inhibitory interneurons is increasingly recognized, as summarized in the recent reviews of Kim and Augustine (2021) and Halverson et al. (2022).

Based on their location, classically three major sets of inhibitory interneurons may be distinguished: those in the molecular layer (MLIs), those in the granule cell layer and the adjacent Purkinje cell layer (“Golgi cells”), and those in the cerebellar nuclei (Ramon y Cajal 1909). As detailed in Schilling et al. (2008), the term “Golgi cell” is now reserved for two major sets of inhibitory interneurons in the granule cell layer, which are clearly distinct from inhibitory interneurons resident in the very upper granule cell layer and within the Purkinje cell layer, i.e., Lugaro cells (for a recent reference, see Miyazaki et al. 2021), globular cells (which were originally considered as part of an extended set of Lugaro cells; Laine and Axelrad 2002), and candelabrum cells (Laine and Axelrad 1994). These latter three types of cells are also referred to as “Purkinje cell layer-associated” inhibitory interneurons (“PLIs”). Some of the many questions still out about cerebellar inhibitory interneurons can now be approached based on single-cell gene expression studies.

### Inhibitory interneurons in the Purkinje cell layer (PLIs) and Golgi cells

A first issue informed by scRNA data relates to the nature and wiring of **candelabrum cells**. Based on the work of Kozareva et al. (2021), Osorno et al. (2022) finally, some 28 year after the first description of these cells (Laine and Axelrad 1994), were able to unravel their synaptic wiring and fundamental aspects of their physiology. They verified that candelabrum cells are, as reported by Simat et al. (2007), purely GABAergic (i.e., not using glycine as a (co-)transmitter), that they receive glutamatergic, excitatory input from mossy fibers and granule cells, and that they are strongly inhibited by Purkinje neurons and also receive inhibitory input from molecular layer inhibitory interneurons. Candelabrum cells, in turn, target and inhibit primarily inhibitory interneurons in the molecular layer (Osorno et al. 2022).

Yet these observations also bring up a new question: As Fabrice Ango’s group impressively showed, neuropilin-1 (Nrp1) mediates the attraction of MLI cell axons towards semaphorin-3a (Sema3a) expressing Purkinje neurons and is also instrumental for attachment of basket-forming axons with the neurofascin-186-expressing axon-initial segments of Purkinje cells. Importantly, their data also reveal that the interactions of Nrp1 with Sema3a and NF186 are mediated by distinct domains of Nrp1 (Telley et al. 2016). How then can we explain why candelabrum cells, which also express high levels of Nrp1, do not target PC axonal initial segments, but rather preferentially innervate MLIs (Osorno et al. 2022)? Taking a methodological perspective, two scenarios come to mind: First, the temporal sequence of expression of NF186, Sema3a, and Nrp1 in Purkinje cells, MLIs, and candelabrum cells could favor innervation of nascent MLIs by candelabrum cells before Purkinje cells, in turn, become attractive for MLIs. To test this, it will be necessary to discern the cell types involved early on, arguably during the late embryonic stage when Purkinje cells physiologically change from multi-dendritic cells to their typical bipolar configuration (Armengol and Sotelo 1991; Schilling et al. 1991a). The second scenario pinpoints one of the limitations of scRNA data currently available for the developing cerebellum. We do not yet know whether MLIs targeting the Purkinje cell axon initial segment and candelabrum cells express identical isoforms of Nrp1. To wit, several splice variants of Nrp1 have been identified (Hendricks et al. 2016; Cackowski et al. 2004; Huang et al. 2019) that predictably affect their interactions with both Nrp1 and Sema3a. And while it is known that differential splicing of Nrp1 occurs in the murine cerebellum (Mazin et al. 2021, and the web-site associated with this publication), we do not yet know whether candelabrum cells and MLIs express distinct splice variants.

**Fig. 4:**
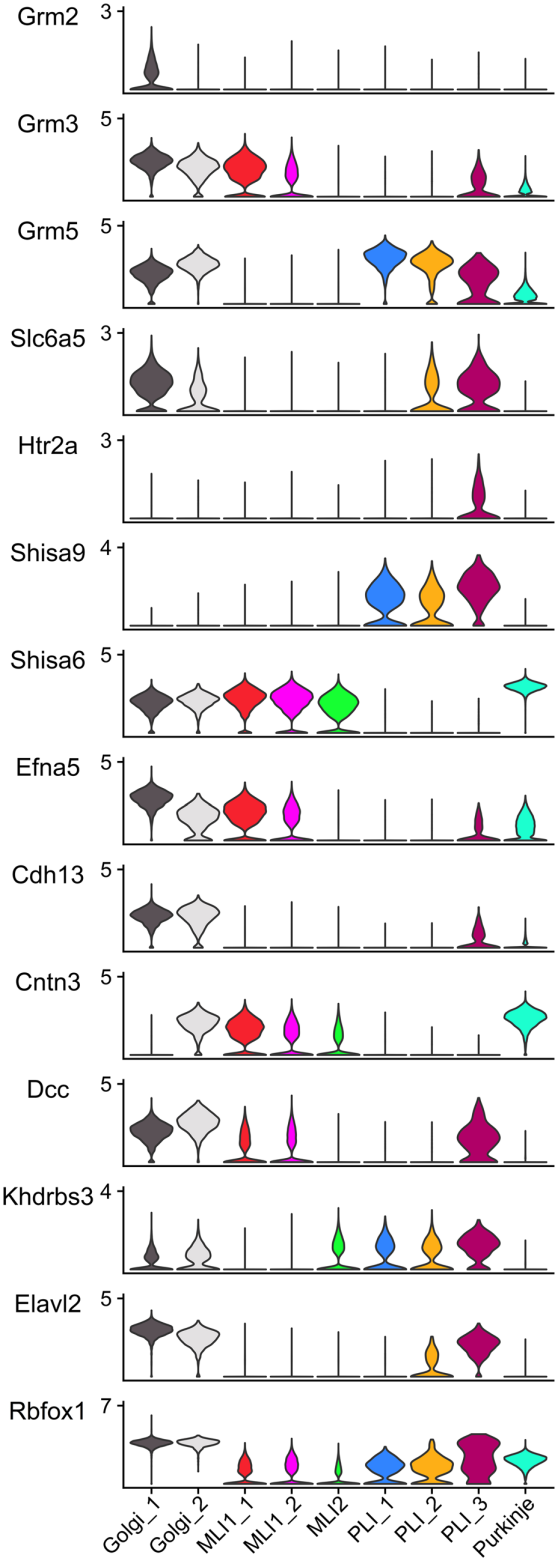
A few examples of genes differentially expressed in various subsets of cerebellar cortical inhibitory interneurons, and, for comparison, Purkinje cells. Data from Kozareva et al. (2021). Cell types were identified as described by Osorno et al. (2022). Two sets of Golgi cells may be distinguished, which correspond to cells positive or negative for the metabotropic glutamate receptor type 2 (Grm2) as delineated by Simat et al. (2007). The subdivision of the molecular layer interneurons (MLI1_1, MLI1_2, MLI2) and Purkinje cell layer interneurons (PLI1, PLI2, PLI3) follows the results reported by Kozareva et al. (2021) and Osorno et al. (2022). As detailed by the latter authors, PLI1 cells correspond to candelabrum cells, PLI2 cells represent globular cells, and PLI3 cells are Lugaro cells. Genes chosen to exemplify differences between various types of inhibitory interneurons include those coding for metabotropic glutamate receptors 2, 3, and 5 (Grm2, Grm3, Grm5), the glycine neurotransmitter transporter Slc6a5, the serotonin receptor Htr2a, and two genes encoding AMPA-receptor interacting proteins (Shisa6, Shisa9); further, four genes coding for proteins associated with cell adhesion, migration, and/or neuritogenesis (Efna5, Cdh13, Cntn3, and Dcc) and three splicing-associated proteins (Khrdbs3, Elavl2, and Rbfox3) are shown

As summarized previously (Schilling et al. 2008), Lugaro and globular cells can be clearly distinguished from Golgi neurons based on their dendritic spread and by the projection of their axons into the molecular layer (see also Fig. 1). In contrast, the targets of Golgi cells are confined to the granule cell layer. Moreover, the size and shape of the somata of Lugaro and globular cells and their location in the uppermost ranges of the granule cell layer facilitate their diagnosis. Analysis of genes expressed by these cells (Kozareva et al. 2021) underlines their character distinct from Golgi cells, from candelabrum cells, and from cells of the lower molecular layer and may direct their further physiological characterization. A few examples are shown in Fig. 4; for further details, see Osorno et al. (2022).

The serendipitous generation of a mouse line in which a substantial fraction of Lugaro cells are tagged by a transgene allowed Miyazaki et al. (2021) to further clarify their wiring and transmitter phenotypes. Thus, these authors could show that Lugaro cells, beyond their known innervation by Purkinje cells and by serotoninergic fibers, receive inputs from climbing fibers, mossy fibers, granule cells, and Golgi cells. Lugaro cells also innervate each other reciprocally. An intriguing result of this study is that the somato-dendritic compartments of Lugaro cells aligned with cerebellar compartments defined by expression of zebrin II (aldolase C; Ahn et al. 1994; Consalez and Hawkes 2013). Lastly, the data of Miyazaki et al. (2021) also suggest that about two-thirds of the Lugaro cells [expressing the identifying transgene] are mixed GABAergic/glycinergic, with most of the rest being exclusively GABAergic.

As for Lugaro and globular cells, scRNA analysis of Golgi cells fully confirms the classification scheme given by Simat et al. (2007). Specifically, the two Golgi cell clusters identified by Kozareva et al. (2021) align with Golgi cells expressing or devoid of the mGluR2 receptor as defined by Simat et al. (2007). While Golgi neurons are traditionally viewed as an electrophysiologically quite homogeneous group of cells (e.g., Geurts et al. 2003; Prestori et al. 2019), the recent observation that they may release, in a regulated manner, GABA and glycine at variable ratios (Dumontier et al. 2023), added a novel, dynamically tunable degree of variability of these cells. Gene expression data (Kozareva et al. 2021) further broaden the perspective granted by Simat et al. (2007) and Dumontier et al. (2023) and reveal additional heterogeneity of these cells. Thus, several of the genes expressed in a differential, if graded fashion more or less along the axis defined by the two Golgi cell clusters mentioned above have been associated with (synaptic) cell adhesion, axonal development, extracellular matrix structure, and/or code for ion channels (e.g., Cntn3, Cntn4, Cntn5, Cntn6, Kirrel3, Efna5, Epha4, Ebf3, Vwc2, Kcnd2). These differences beg the question whether they relate to morphologically discernable characteristics (i.e., wiring preferences) or synaptic receptor localization/efficiency. However, their physiological significance awaits study.

**Fig. 5:**
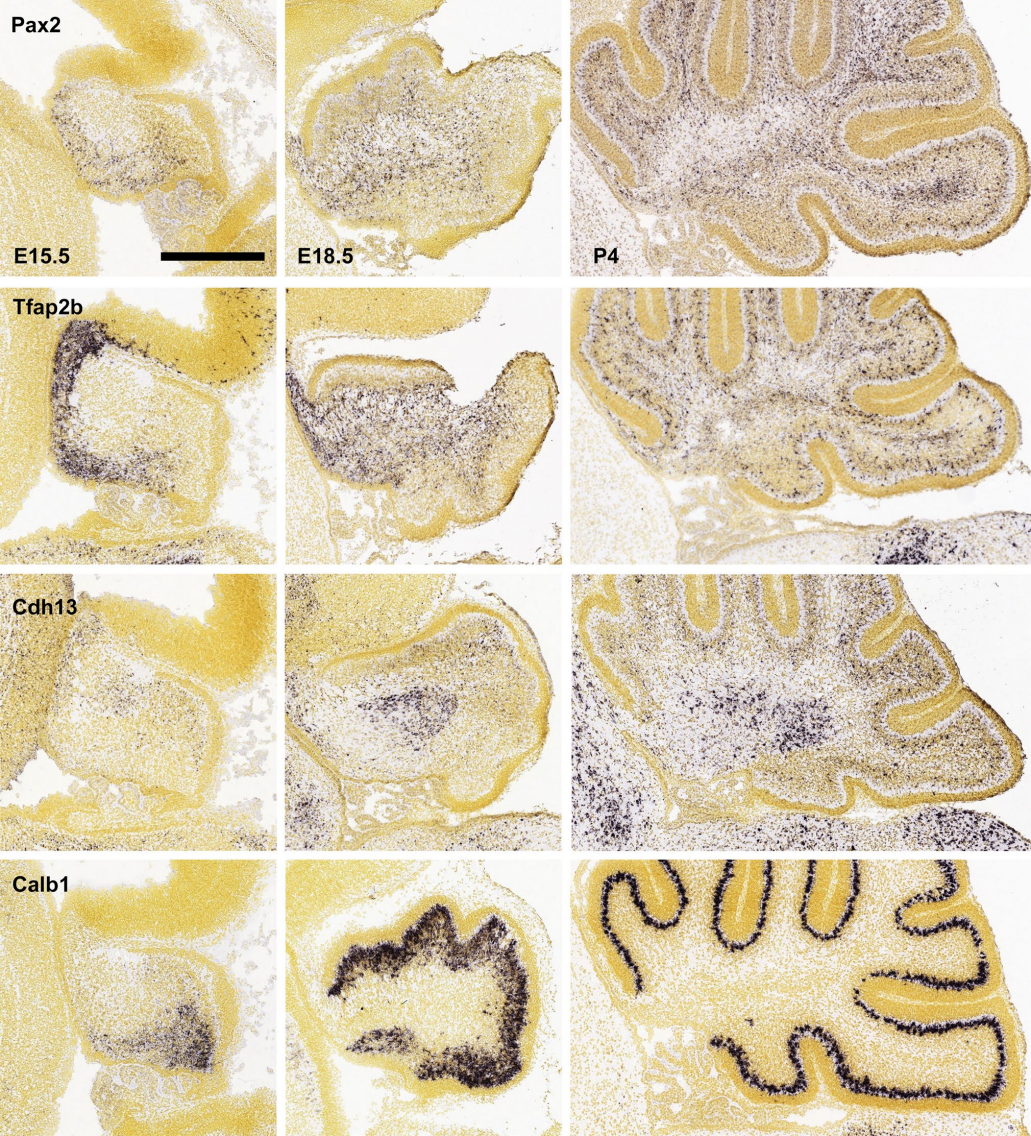
Spatial distribution of precursors of cerebellar (cortical) inhibitory interneurons as indicated by the expression of Pax2, Tfap2a, and Cdh13 as seen in sagittal sections of mice at embryonic days 15.5 and 18.5 and at postnatal day 4. The distribution of postmitotic Purkinje neurons stained for calbindin D-28K (Calb1) is shown to facilitate orientation. Note that Pax2 is expressed only as prospective inhibitor interneurons go through their last mitosis and does not stain cells in the ventricular epithelium. Also, this gene is rapidly downregulated as molecular layer interneurons enter the nascent molecular layer and thus, in contrast to a GFP-transgene based on the Pax2 gene (Wefers et al. 2017), does not allow us to monitor dispersal of interneurons in the molecular layer. Staining for Tfap2a reveals a similar distribution, and like Pax2 reveals the transit of precursors of inhibitory interneurons of the molecular layer through the nascent white matter at E18.5 and P4. Lastly, staining for Cdh13 allows us to follow the spatiotemporal distribution of Golgi cells (and Lugaro cells; cf. Fig. 4). Note that these cells can first be seen anteriorly, and that they can no longer be seen in the nascent white matter at P4, when they are restricted to the nascent internal granule cell layer. Strongly Cdh13-positive cells can also be seen in the nascent cerebellar nuclei at all ages shown. Rostral is to the left, dorsal to the top; scale bar = 0.5 mm. All images were taken from the Allen Brain Atlas (Lein et al. 2007)

The function of the cell adhesion molecule cadherin 13 (Cdh13), which in the cerebellar cortex is strongly expressed in Golgi neurons (e.g., Schilling and Oberdick 2010), and perhaps also in Lugaro cells (see Fig. 4), was recently studied by Tantra et al. (2018). These authors ablated Cdh13 expression from the majority of Golgi cells using a Slc6a5-driven Cre construct. This resulted in decreased expression of the mRNA coding for GABA-synthesizing enzyme, Gad1, in Golgi cells, and a selective reduction in the strength or numbers of inhibitory synapses, but not excitatory synapses, formed onto Golgi cells. It may be added that the knockout strategy chosen by these authors is predicted to eliminate Cdh13 expression in Lugaro cells as well—if the expression of Cdh13 in these cells as indicated in Fig. 4 stands scrutiny. Of note, one Lugaro cell targets some 100 Golgi cells (Dieudonne and Dumoulin 2000). Moreover, the knockout strategy chosen should also abrogate Cdh13 expression by inhibitory neurons of the cerebellar nuclei, notably of the cluster i3 as defined by Kebschull et al. (2020), which in turn project to the cerebellar cortex and innervate Golgi cells. Indeed, the subset of Golgi cells that are targeted by these nuclear neurons do not express Slc6a5 (Ankri et al. 2015), and should thus not be directly affected by the knockout strategy employed by Tantra et al. (2018). It is not known whether these two populations of Golgi cells were differently affected by the ablation of Cdh13. As already mentioned above, available data also suggest that cells from the pc4 region of the cerebellar ventricular epithelium contribute to the generation of Golgi cells. The spatiotemporal pattern in which Cdh13-positive cells may be observed in the developing cerebellum further begs the question whether at least some of these cells populate the nascent cortex in an anterior–posterior direction, i.e., in a direction opposite to the main direction of Purkinje cell dispersal (Fig. 5 bottom row).

Lastly, Golgi neurons have recently also been identified as the probable source of GABAergic signaling that shapes the development of granule cell dendrites (Dhar et al. 2018).

### Diversity of MLIs—basket and stellate cells

Traditionally, inhibitory interneurons resident in the molecular layer (MLIs) are classified as either basket or stellate cells, distinguished by the fact that the axons of the latter do not contact Purkinje cell somata (Smirnow 1897; Ramon y Cajal 1909, p. 19–32). How then do these morphological differences relate to the distinct genetic signatures obtained for molecular MLIs (Schilling and Oberdick 2010; Kozareva et al. 2021)? To address this issue, it is important to recall that the classification of MLIs in the upper molecular layer (ML) as stellate cells and those in the lower ML as basket cells is at best a textbook simplification. Arguably, it can be traced back to a summary description of Ramon y Cajal, who stressed the star-like arrangement of the dendrites of the smaller MLIs in both the upper and lower ML, and referred to the larger MLIs in the lower ML as “*cellules étoilées profondes ou encore cellules à corbeilles*” (Ramon y Cajal 1909, p 19).

Indeed, it is now well established that MLIs throughout the molecular layer may impress as “star-like,” which refers to the structure of their dendrites (cf. Ramon y Cajal, cited above). Yet it has also been established that axons of inhibitory interneurons from all ranges of the molecular layer do contribute to the basket-like ensheathing of Purkinje cell somata and axon initial segments. The fine axons of basket cells resident in the upper molecular layer was beyond the optical resolution available to Ramon y Cajal, who nevertheless pointed out that these cells had axons directed towards the Purkinje cell layer (Ramon y Cajal 1909, p31). A first quantitative study to assess the morphologic variability of MLIs and its relation to the position of their somata in the molecular layer was presented some 25 years ago by Sultan and Bower (1998). While one may wonder whether this seminal study may have been under-powered given its small sample size, its key findings were recently fully confirmed by Wang and Lefebvre (2022), who studied a much larger data set. Their analysis again shows that basket cells may be found throughout the molecular layer, with a preference for basket cells to be positioned closer to the Purkinje cell layer (*r*^2^ = 0.30 for the distance of MLI somata and their axons contacting Purkinje cell somata). One implication of the findings of Wang and Lefebvre (2022) is that the preferential expression of a set of genes in MLIs in the lower but not the upper molecular layer (for a list of genes, see Schilling and Oberdick 2010; see also Sergaki et al. 2017 for Ret) cannot be considered markers of basket cells. Rather, the quest for the developmental and functional implication(s) of these differences in gene expression is still open.

Combining their extensive morphometric data with the gene expression data reported by Kozareva et al. (2021), Wang and Lefebvre (2022) concluded that morphologically unambiguously identified basket and stellate cells do not align with the genetically defined clusters MLI1 and MLI2 reported by Kozareva et al. (2021). In an apparent contradiction, Osorno et al. (2022) state that subclustering of MLI1 cells allows one to distinguish basket and stellate cells. As these authors write, “MLI1_1 and MLI1_2 correspond to basket cells and stellate cells, respectively, which have distinctive morphologies, synapse distribution and locations in the molecular layer.” Yet the question of how they arrive at this summary statement without morphometric identification of the cells within these subclusters remains open.

### Inhibitory interneurons of the cerebellar nuclei

Of all cerebellar inhibitory interneurons, those in the cerebellar nuclei are arguably the most enigmatic. Pax2-positive cells have been described in the developing and adult cerebellar nuclei (Maricich and Herrup 1999; Weisheit et al. 2006; Leto et al. 2009; data from the website associated with Kebschull et al. 2020), and by analogy with Pax2-expressing cells in the cerebellar cortex, these cells are generally thought to be interneurons. Yet it should be pointed out that other than their rather late generation (final mitosis), which contrasts with the early formation of established nuclear projection neurons (cf. Kebschull et al. 2023), no independent data supporting the interneuronal character of these cells are available. As already mentioned above, at least a subset of the GABAergic projection neurons connecting, across the midline, the fastigial nuclei also expresses Pax2 (Gomez-Gonzalez and Martinez-Torres 2021). Further, the inhibitory nuclear cells targeting a subset of Golgi cells identified by Uusisaari’s group (Ankri et al. 2015) are presumably also derived from Pax2-positive precursors (see also Kebschull et al. 2023 for further discussion). Together, either these results challenge the view that cerebellar Pax2-positive precursors exclusively give raise to inhibitory interneurons, or we may have to broaden our definition of what may be considered an interneuron (in the cerebellar nuclei).

## How do cerebellar inhibitory interneurons differentiate?

It is our current understanding that Pax2-positive precursors diversify in response to local signals received while migrating from the ventricular epithelium through the deep cerebellar mass and nascent white matter to their target territories (Leto et al. 2010). The striking correlation between when these cells go through their last mitosis and their final fate (Leto et al. 2009) may be taken as an exemplary realization of the old saying that correlation does not imply causation. As established in elegant transplantation studies by Ketty Leto and the late Ferdinando Rossi (Leto et al. 2006, 2009), the neurochemical and morphological specification of these cells occurs in response to local signals that Pax2-positive precursors are exposed to in the dynamically changing environment they find themselves in as cerebellar development proceeds. We owe these authors the view that the nascent white matter of the cerebellum forms an “instructive niche” (Leto et al. 2010) for cerebellar inhibitory interneurons. Unfortunately, our knowledge about the physical character of signals acting on immature precursors of inhibitory cerebellar interneurons is still quite limited. Some progress has been made for inhibitory interneurons eventually populating the molecular layer (MLIs), which are experimentally more easily accessible than the predominantly prenatally developing interneurons of the cerebellar nuclei and the granule cell layer.

### Generating a sufficient complement of inhibitory interneurons, at appropriate proportions

In contrast to developing cerebellar GABAergic projection neurons, precursors of inhibitory interneurons do not leave the cell cycle as they emigrate from the ventricular epithelium, and they continue proliferating extensively in the cerebellar deep mass and nascent white matter. Proliferation in the cerebellar ventricular zone ceases around embryonic day 17.5 (Haldipur et al. 2022). Yet the bulk of precursors for inhibitory interneurons become postmitotic only postnatally, as indicated by their expression of Pax2 (Weisheit et al. 2006). Combining these data, we may estimate that more than 90% of all prospective inhibitory interneurons go through their last mitosis in the nascent deep cerebellar mass/white matter.

Two signaling molecules, sonic hedgehog (Shh) and glial cell-derived neurotrophic factor (Gdnf; which despite its name may also be synthesized by neurons), have been identified supporting the proliferation of interneuronal precursors in the nascent white matter and thus contribute to the exact, species-specific (Lange 1975; see also Ito 1984, p 75 and p 106 f) numerical matching of inhibitory interneurons, Purkinje cells, and granule cells.

Sonic hedgehog stimulates proliferation of both Ptf1a-positive interneuronal precursors in the cerebellar ventricular epithelium and those in transit through the white matter (Huang et al. 2010; Fleming et al. 2013; de Luca et al. 2015). In 2013, Fleming et al. showed that Shh, which drives proliferation of Pax2-positive cells in the nascent white matter, is produced by Purkinje cells. Thus, Purkinje cells use the same signaling molecule by which they *locally* regulate the expansion of granule cell precursors (Smeyne et al. 1995) to adjust the generation of inhibitor interneurons at some distance. The conceptually attractive idea that Purkinje cells might specifically regulate the proliferation of inhibitory interneurons that eventually will directly interact with them has so far not been tested. The data of de Luca et al. (2015) further suggest that developing inhibitory interneurons might still be responsive to Shh as they leave the cell cycle, implying that Shh could also be involved in the differentiation of MLIs. This idea seems also supported by the observation that animals in which the Shh signal transducer Smo was knocked out in Ptf1a-positive cells showed a reduced proliferation of these cells and a quite specific loss of MLIs in the upper molecular layer (Li et al. 2022).

Conversely, elimination of Shh signal transduction by targeting even earlier stem cells, which give rise to both astroglial cells and Pax2-positive cells (and presumably NG2-cells), was reported to affect numbers of interneurons in the MLI and the granule cell layer (Fleming et al. 2013). As for the development of cerebellar inhibitory interneurons, these two targeting strategies to abrogate Shh-sensitivity differ in at least two significant ways. First, they eliminate Shh-signaling at quite different developmental stages. Second, as the approach chosen by Fleming et al. (2013) also affects glial cells, it may also impinge on the microenvironment, the “developmental niche” (Leto et al. 2010) in which Pax2 cells differentiate.

Sergaki et al. (2017) reported that the development of MLIs is also dependent on GDNF/Ret/Gfra1 signaling, and that ablation of either Ret or Gfra1 (in Ptf1a lineage cells; i.e., all cerebellar inhibitory interneurons, Purkinje cells, and also nuclear inhibitory neurons except the cells described by Bagnall et al. 2009; cf. above) resulted in a specific loss of some 25% of MLIs. Unfortunately, the mechanistic interpretation of these findings is fraught with discrepancies between the expression of the Ret^GFP^-knock-in construct used to identify Ret-expressing cells and that of cognate Ret, as may be taken, say, from data in the Allen Brain Atlas (Lein et al. 2007) or as documented in recent single-cell gene expression studies (Kozareva et al. 2021; Vladoiu et al. 2019; Khouri-Farah et al. 2022). Thus the (strong) expression of Ret^GFP^ in Pax2-positive cells of the nascent white matter reported by Sergaki et al. (2017) cannot be seen for cognate Ret; conversely, cognate Ret is strongly expressed in large-bodied cells in the granule cell layer (presumptive Golgi cells), whereas Ret^GFP^ was not found in these cells. Indeed, single-cell analysis of a total of 2236 interneuronal precursors obtained from p4, p8, and p12 old animals, i.e., in the time window during which most MLIs are formed and enter the molecular layer (Leto et al. 2009; Wefers et al. 2017), showed that ~ 6.6% expressed Ret, ~ 15.9% expressed Gfra1, but only 15 out of 2236 cells (~0.7%) coexpressed Ret and Gfra1 (gene expression data from Kozareva et al. 2021). Consistent percentages were obtained when this analysis was limited to the 1069 Pax-2 positive cells in the sample (4.4, 24.6, and 0.8%).

An alternative explanation of the data reported by Sergaki et al. (2017) might be that Gdnf impinges on the development of cerebellar inhibitory interneurons by acting on Gfra1 in association with Ncam (Paratcha et al. 2003; Ibanez et al. 2020), which is widely and rather strongly expressed in virtually all precursors of cerebellar cortical interneurons. This so far hypothetical Ncam-assisted Gdnf signaling in MLIs does not exclude an effect of Ret, as described. However, given the very sparse expression of Ret in precursors of MLIs, Ret-mediated signaling can hardly be cell-autonomous, as postulated, based on the mistaken notion “that no other cerebellar cell type apart from MLIs expresses Ret” (Sergaki et al. 2017). As documented in the Allen Brain Atlas and in the online data accompanying the recent paper by Kebschull et al. (2020), Ret is also expressed in cerebellar nuclei. Further, any attempt to adjust the intriguing model suggested by Sergaki et al. (2017) needs to account for the observation that, at least in adult murine cerebella, gene expression data suggest that Gdnf is much more strongly expressed in Golgi cells than in Purkinje cells (Kozareva et al. 2021). Lastly, Ret is also involved in other signaling pathways, notably that of ephrins and their receptors (Bonanomi et al. 2012; Lisabeth et al. 2013), some of which are prominently expressed in nascent inhibitory interneurons (cf. Liebl et al. 2003; and data of Kozareva et al. 2021).

Be that as it may, interference with Gdnf/Ret/Gfra1 signaling in the Ptf1a lineage resulted in a specific loss of about 25% of MLIs, obviously without affecting numbers of other inhibitory neurons derived from Ptf1a-positive precursors (Sergaki et al. 2017).

A loss of about 50% of parvalbumin-positive MLIs, but not of neurogranin-positive Golgi neurons, was also observed in Prdm13-deficient mice (Whittaker et al. 2021). The authors of this publication did not probe whether the loss of MLIs was related to their position (depth) in the molecular layer, though their data show that both morphologically intact basket and stellate cells, discernible by their axonal projections, were still found in Prdm13-null animals. Also, the effects of Prdm13 ablation on the some 20% of neurogranin-negative Golgi cells and PLIs was not tested. Still, the findings of Whittaker et al. (2021) are consistent with the notion that reduced generation of Pax2-positive precursors of inhibitory interneurons leads to a preferential deficit in the upper regions of the cerebellar cortex. Lastly, a preferential loss of later-born inhibitory interneurons (i.e., of those that normally settle in the upper molecular layer) was also observed in mice lacking Ascl1 in the cerebellum (Sudarov et al. 2011).

None of the conditions just described—i.e., signaling via Shh and Gdnf, or mutations in Prdm13 and Ascl1—may be presumed to act selectively on precursors of cerebellar inhibitory interneurons, and thus probably also impinge on the “instructive niche” these cells find themselves in. Yet a common aspect of these various conditions is that they all preferentially affect the development of cerebellar cortical inhibitory interneurons in the upper (outer) cerebellar cortex/upper molecular layer. This is strongly reminiscent of the failed generation of MLIs of the upper molecular layer after ablation of cyclin D2 described in a classical study by Huard et al. (1999). Thus, five genetically and mechanistically distinct conditions that all impede the generation of a normal complement of cerebellar inhibitory interneurons consistently result in a preferential deficit of inhibitory interneurons in those cortical areas that, during normal development, are reached and settled last.

**Fig. 6:**
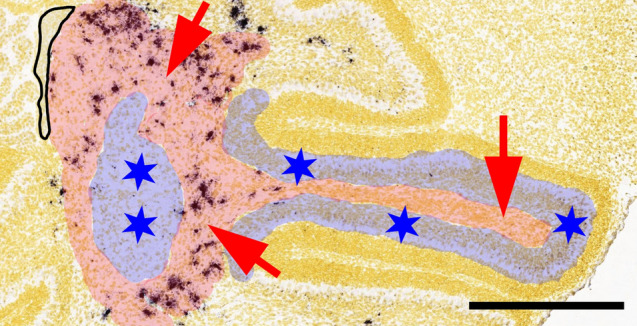
A revised version of the “instructive niche model.” This image depicts part of a developing murine cerebellum at postnatal day 4. Part of the ventricular epithelium from which precursors of inhibitory interneurons originate is still visible and delineated by a black line at the left margin of this image. The deep cerebellar mass/nascent white matter where immature inhibitory interneuronal precursors are presumed to be programmed towards their final fate according to the original version of the “instructive niche model” is colored in red. Cerebellar nuclear and cortical areas suggested to cause the final differentiation of inhibitory interneurons according to the revised model proposed here are colored in blue. Red arrows symbolize (so far unidentified) instructive signals in the nascent white matter, and blue stars indicate that territories settled by differentiating interneurons lose their attraction for later-arriving inhibitory interneuronal precursors as suggested by the present model. The background image was obtained from the Allen Brain Atlas and shows a few oligodendrocytes expressing MBP, one of the cell types that might impinge on late-, but not on early-generated inhibitory interneuronal precursors (cf. also Groteklaes et al. 2020). Scale bar ~ 0.4 mm

A parsimonious and integrative explanation for these observations might be as follows (Fig. 6): Cerebellar nuclei, the granule cell layer, and the lower and upper molecular layer all form attractive targets for prospective inhibitory interneurons. Yet as they get settled by interneuronal precursors, and as these start to differentiate, their attractiveness wanes, or they possibly become even repulsive for immature interneuronal precursors, which consequently continue to migrate towards more distant but still attractive regions. If too few immature cells are formed, the regions farthest off would remain unsettled. Following experimental ablation of granule cell precursors and the consequent reduction of parallel fibers, immature MLIs still accumulate next to the cerebellar surface but fail to differentiate and to disperse (Cadilhac et al. 2021). This underscores the fact that differentiation of inhibitory interneurons is crucial to abrogating the unknown attractive signal for prospective MLIs. Finally, the observation, made 30 years ago (Napieralski and Eisenman 1993; see also Napieralski and Eisenman 1996; Hamre and Goldowitz 1996), that immature precursors of inhibitory interneurons still migrate beyond the forming Purkinje cell layer in the anterior cerebellum of meander tail mice suggests that granule cells and parallel fibers are not needed to make the region beyond Purkinje cell somata an attractive target for immature inhibitory interneurons. The final fate of cerebellar inhibitory interneurons would then be defined by signals received once they settled.

The scenario just sketched is consistent with the “instructive niche” model (Leto et al. 2010) introduced above and, importantly, with the experimental data that led to its formulation. However, it somewhat shifts the perspective given in its original formulation, where the focus was on the changing microenvironment that precursors of inhibitory interneurons would be exposed to in the nascent white matter (Leto et al. 2009, 2010). Here, I propose taking into account that territories not yet occupied by differentiating inhibitory interneurons also change substantially as cerebellar development proceeds. Thus, in a young animal, with only a few interneurons formed, there is much more "land" to be settled than in an older host, which has almost completed its endogenous program of interneuron generation and dispersal.

### Migration and local integration of cerebellar inhibitory interneurons

As indicated above, most precursors of cerebellar inhibitory interneurons go through their last mitosis in the nascent white matter, and it is only then that they start to express Pax2 and can be unambiguously identified. This implies that, so far, the initial dispersal of these cells into the cerebellar anlage can only be inferred from the spatiotemporal pattern of the appearance of Pax2-positive cells (Fig. 5).

There is some rather anecdotal data depicting the movement of Pax2-positive cells along the folial axes within the nascent white matter as the cerebellar cortex acquires its characteristic folds (see supplementary materials to Hecker et al. 2008, and also to Wefers et al. 2017). However, we are currently ignorant about distances covered this way, and about numbers of cells that do so. We are also completely ignorant about what triggers the eventual transit of these cells into nascent cerebellar nuclei or the emergent cortex, at appropriate locations, and at appropriate numbers.

Conversely, the migration of Pax2-positive cells in the cerebellar cortex, and notably in the molecular layer, has been studied using a variety of model systems. Cameron et al. (2009) used cultures of sagittally oriented slices from 5- to 14-day-old mice and described the ascent of prospective MLIs through the cerebellar cortex as occurring in a predominantly radial or oblique direction. After reaching the border of the external granule cell layer, the cells switched and migrated tangentially. Of note, in sagittally oriented slices, “tangential” indicates a direction perpendicular to the (in situ) orientation of parallel fibers. This mode of migration was observed within the first 2 days of cultivation. Subsequently, cells were observed migrating again in a radial direction, but inward, until finally they moved again tangentially and started to extend dendrites, which were preferentially oriented parallel to the Purkinje cell layer.

The tangential migration described by Cameron et al. (2009) was not observed in acute slices analyzed by Cadilhac et al. (2021). Rather, these authors noted a tangential migration in the mediolateral direction, i.e., along the developing parallel fibers of granule cells leaving the external granule cell layer. Cadilhac and associates plausibly discuss this discrepancy with the inevitable cellular and molecular reorganization that occurs in cultured slices over time, notably that of granule cell axons and the expression of the cell adhesion molecule Tag-1 by these cells.

While the data of Cadilhac et al. (2021) greatly contribute to our understanding of the migration of MLIs, one caveat nevertheless seems appropriate. These authors suggest that the tangential mode of migration they observed might be specific for later-arriving MLIs in the upper molecular layer (to which they refer as stellate cells). However, it should be noted that they did not specifically exclude such a tangential migration mode for early-arriving MLIs. Such an analysis might in fact be difficult for two technical reasons. First, up to postnatal day 3—the time point at which these authors monitored migration in the lower molecular layer—only about 10–20% of all Pax2-expressing cells have formed, and the behavior of the few cells that by then have arrived at the border of the external granule cell layer may have been missed. This problem may have been further aggravated by the fact that the Gad67-Gfp transgene Cadilhac and associates used to identify MLIs is developmentally regulated and, in the cortex, its expression “stabilized during the second postnatal week” (Chattopadhyaya et al. 2004). Moreover, given that in the cortex this transgene labeled only 50% of parvalbumin-positive interneurons, verification of its expression in early developing MLIs entering the molecular layer would be highly desirable to substantiate the different migration modes for differentiating MLIs in the lower versus the upper molecular layer, as suggested by Cadilhac et al. (2021).

Like Cadilhac et al. (2021), Wefers et al. (2017) used two-photon imaging to assess migration of prospective MLIs in acute slices of the early postnatal cerebellar anlage (P7–P9). Migrating precursors of MLIs were identified based on their expression of a Pax2-GFP transgene (Pfeffer et al. 2002; Weisheit et al. 2006). Technically, this study also differed from the two studies just discussed in that it allowed for monitoring of cell positions with a much higher temporal resolution [one image every 42 s; in contrast to one image every 10 min (Cadilhac et al. 2021) or even every 30 min (Cameron et al. 2009)], and indeed in three dimensions. The key finding of this study was that Pax2-expressing precursors of MLIs receive synaptic input as they migrate into the molecular layer, and that abrogation of this input disturbed their directional migration. This effect contrasts with the previously known effects of changes in the ambient levels of neurotransmitters or global transmitter receptor blockade in the developing nervous system, which predominantly affects speed (for further details and references, see discussion in Wefers et al. 2017). These apparently conflicting observations may be readily reconciled considering that fluctuations in ambient transmitter levels or general receptor blockade result in non-localized, or isotropic signals on individual cells, whereas synaptic signals are highly localized and provide an anisotropic stimulus.

While Wefers et al. (2017)—including the present author—considered parallel fibers as a rather unlikely source of synaptic input for migrating MLIs, this view may need revision given the results recently reported by Park et al. (2019). In their elegant study, these authors selectively abrogated synaptic/secretory activity of subsets of granule cells in the developing cerebellum. Consequently, the nascent molecular layer comprised stacked territories of active and muted parallel fibers. In these animals, MLIs were misplaced, a finding that corroborated the significance of synaptic activity for MLI migration described by Wefers et al. (2017). It may be added that the abnormal development of Purkinje cell dendrites also seen in this study could be taken as an independent indicator of parallel fiber silencing (cf. Schilling et al. 1991a). Importantly, the (transient) lack of parallel fiber activity in these mice resulted in persistent motor deficits.

Lastly, the observation that immature MLIs were found expressing rather high levels of immediate early genes, notably Fos (Kozareva et al. 2021; this finding is also supported by the data reported by Vladoiu et al. 2019), is consistent with the significance of external stimuli operative during the development and settling of these cells. However, it should be stressed that the sensitivity of Fos to a broad spectrum of external stimuli (e.g., Schilling et al. 1991b; Smeyne et al. 1992) does not allow us to draw any conclusions as to the nature of those acting on developing MLIs, which indeed may include stimuli due to tissue preparation.

If physiological, the activation of immediate-early genes like Fos pinpoints generalized changes in gene expression of stimulated cells. Conversely, the synaptic input as identified by Wefers et al. (2017) is predicted to result in highly localized effects. It is well established that neurotransmitter-mediated ionic currents or second messenger fluctuations may (transiently) alter migratory activity or axonal steering by modulating the sensitivity of growth cones/leading processes to both diffusible and localized migratory cues (e.g., Hong et al. 2000; Ming et al. 1997). Mechanistically, this may be realized, for example, by altering translation of localized mRNAs (e.g., Holt and Schuman 2013; Perez et al. 2021; Lin and Holt 2007), or by depolarization-induced growth cone collapse (e.g., Xu et al. 2019), or more generally, by interfering with second messenger networks integrating extracellular cues that orient migrating cells or sprouting axons in the developing brain (Baudet et al. 2023).

## Cerebellar inhibitory interneurons and disease

In contrast to the prominent neuropathology and devastating diseases that may be traced to excitatory neuronal lineages of the cerebellum (e.g., Vladoiu et al. 2019; Hendrikse et al. 2022; Luo et al. 2022), to Purkinje cell dysfunction (e.g., Chopra et al. 2018; Jiang et al. 2015), or even to the still somewhat enigmatic cells of cerebellar nuclei (for an extensive review, see Kebschull et al. 2023), our knowledge about disease-related dysfunction of inhibitory interneurons is limited and just about to emerge. Thus, Pilotto and associates (2023) recently reported that Purkinje cell degeneration in spinocerebellar ataxia type 1 (SCA1) can be related to increased activity of molecular layer interneurons, which are characterized by elevated levels of parvalbumin expression and an imbalanced excitatory-to-inhibitory synaptic investment. This, in turn, was related to the expression of increased levels of Frrs1l, a protein implicated in AMPA receptor trafficking. The authors concluded “that circuit-level deficits upstream of [Purkinje neurons] are one of the main disease triggers in SCA1” (Pilotto et al. 2023).

As already discussed above, reduced generation of Pax2-positive precursors of inhibitory cerebellar interneurons has also been observed in humans with mutations of Prmd13 (Whittaker et al. 2021). Whether and how this lack of a full complement of inhibitory interneurons might contribute to the severe (neuro-)pathology of the affected subjects, which also suffer from Purkinje cell dysfunction (Coolen et al. 2022), remains to be seen.

Lastly, it should be recalled that the development of human excitatory cerebellar cell lineages has recently been shown to differ significantly, and in a disease-relevant way, from that of mice, and indeed other nonhuman primates (Haldipur et al. 2019). These findings beg the question whether similar species differences exist for inhibitory cerebellar interneurons, the numbers of which, like those of excitatory granule cells, scale positively with those of Purkinje cells as brains get larger (Lange 1974; Ito 1984, p 74; Korbo et al. 1993; Huang et al. 2014).

## (Conclusions and) perspectives

To conclude, the integration of an increasing number of single-cell-based gene expression studies with classical developmental studies, but also with sophisticated imaging, provides a wealth of opportunities to further explore the development and pathology of the cerebellum. Not unexpectedly, the new perspectives these novel data grant come with new and intriguing questions. For example, for a number of genes, scRNA studies and immunocytochemical analyses are at conflict. To name but one example, immunostaining suggests that the splicing factor Rbfox3 (NeuN) is expressed only in excitatory cerebellar cortical cells (Weyer and Schilling 2003), whereas scRNA data indicate that its mRNA is also expressed in inhibitory neurons (see Fig. 4; data from Kozareva et al. 2021). Does this reflect a difference in the sensitivity of these methods, or does this hint at a deeper biological/physiological process, like differential splicing or phosphorylation of the Rbfox3 protein product, which might alter the epitope recognized by its defining antibody (Lind et al. 2005; Maxeiner et al. 2013) and possibly its cellular function? Related questions are of outstanding interest, considering that alternative splicing has been estimated to occur in more than 90% of all multi-exon genes (Wang et al. 2008; Pan et al. 2008), and differential splicing and mRNA editing have been observed for multiple genes crucial for the specific physiology of nerve cells, ranging from pattern formation and wiring (e.g., neurexins; Nguyen et al. 2016; Lin et al. 2023; Hauser et al. 2022) to receptor characteristics and localization (e.g., Wen et al. 2017; Dos Reis et al. 2022; see also Farini et al. 2020 for an example of the significance of splicing for proper cerebellar development). Fortunately, technologies to tackle these issues are being developed at a rapid pace (e.g., Wen et al. 2023; Feng et al. 2021; Benegas et al. 2022; Joglekar et al. 2021; and more recent examples will probably be available as this manuscript goes into print). Simultaneously, the spatial resolution of such techniques and their integration with classical histological approaches advances rapidly (e.g., Baysoy et al. 2023; Alon et al. 2021; Kwon 2023; Shi et al. 2023, and further references there), paving the way for the inclusion of subcellularly localized mRNAs (e.g., Wanner et al. 1997; Crino and Eberwine 1996; Zhang et al. 2008; Holt and Schuman 2013; Mendonsa et al. 2023) in single-cell analyses (for a recent review, see Ament and Poulopoulos 2023). Importantly, technological advances increasingly allow multimodal analyses and integration, at the single-cell level, of mRNA expression with the analysis of transcriptional regulation, protein expression and posttranslational modification, or metabolic activity (see, e.g., Bartosovic and Castelo-Branco 2023; Zheng et al. 2023; Fangma et al. 2023). Together, these technological advances herald even deeper insights into cerebellar development and an informative, exciting future for histochemistry and cell biology in general.

## Footnotes

1 For convenience, I here refer to GABAergic and/or glycinergic neurons as inhibitory, and to glutamatergic neurons as excitatory. Yet it should be remembered that the action of these transmitters is also critically dependent on the receptor complement and/or membrane potential of their target cells, and that GABA can be depolarizing (i.e., excitatory), particularly during development (e.g., Chatterton et al. 2002 and Jean-Xavier et al. 2007 for excitatory actions of GABA and glycine; Nawy 1999, for hyperpolarizing actions of glutamate). I hope the wording used here will not discourage one from considering potential "inverse" effects of transmitters during development, which is particularly well documented for cerebellar "inhibitory" interneurons (Chavas and Marty 2003).

## References

[CR1] Ahn AH, Dziennis S, Hawkes R, Herrup K (1994). The cloning of zebrin II reveals its identity with aldolase C. Development.

[CR2] Alexander T, Nolte C, Krumlauf R (2009). Hox genes and segmentation of the hindbrain and axial skeleton. Annu Rev Cell Dev Biol.

[CR3] Alon S (2021). Expansion sequencing: Spatially precise in situ transcriptomics in intact biological systems. Science.

[CR4] Amat SB, Rowan MJM, Gaffield MA, Bonnan A, Kikuchi C, Taniguchi H, Christie JM (2017). Using c-kit to genetically target cerebellar molecular layer interneurons in adult mice. PLoS One.

[CR5] Ament SA, Poulopoulos A (2023). The brain's dark transcriptome: sequencing RNA in distal compartments of neurons and glia. Curr Op Neurobiol.

[CR6] Ankri L, Husson Z, Pietrajtis K, Proville R, Lena C, Yarom Y, Dieudonne S, Uusisaari MY (2015). A novel inhibitory nucleo-cortical circuit controls cerebellar Golgi cell activity. Elife.

[CR7] Armengol JA, Sotelo C (1991). Early dendritic development of Purkinje cells in the rat cerebellum. A light and electron microscopic study using axonal tracing in 'in vitro' slices. Dev Brain Res.

[CR8] Aroca P, Puelles L (2005). Postulated boundaries and differential fate in the developing rostral hindbrain. Brain Res Brain Res Rev.

[CR9] Bagnall MW, Zingg B, Sakatos A, Moghadam SH, Zeilhofer HU, du Lac S (2009). Glycinergic projection neurons of the cerebellum. J Neurosci.

[CR10] Bartosovic M, Castelo-Branco G (2023). Multimodal chromatin profiling using nanobody-based single-cell CUT&Tag. Nat Biotechnol.

[CR11] Basson MA, Echevarria D, Ahn CP, Sudarov A, Joyner AL, Mason IJ, Martinez S, Martin GR (2008). Specific regions within the embryonic midbrain and cerebellum require different levels of FGF signaling during development. Development.

[CR12] Baudet S, Zagar Y, Roche F, Gomez-Bravo C, Couvet S, Becret J, Belle M, Vougny J, Uthayasuthan S, Ros O, Nicol X (2023). Subcellular second messenger networks drive distinct repellent-induced axon behaviors. Nat Commun.

[CR13] Baysoy A, Bai Z, Satija R, Fan R (2023). The technological landscape and applications of single-cell multi-omics. Nat Rev Mol Cell Biol.

[CR14] Ben-Arie N, Bellen HJ, Armstrong DL, McCall AE, Gordatze PR, Guo Q, Matzuk MM, Zoghbi HY (1997). Math1 is essential for genesis of cerebellar granule neurons. Nature.

[CR15] Benegas G, Fischer J, Song YS (2022). Robust and annotation-free analysis of alternative splicing across diverse cell types in mice. Elife.

[CR16] Bessodes N, Parain K, Bronchain O, Bellefroid EJ, Perron M (2017). Prdm13 forms a feedback loop with Ptf1a and is required for glycinergic amacrine cell genesis in the Xenopus Retina. Neural Dev.

[CR17] Bonanomi D, Chivatakarn O, Bai G, Abdesselem H, Lettieri K, Marquardt T, Pierchala BA, Pfaff SL (2012). Ret is a multifunctional coreceptor that integrates diffusible- and contact-axon guidance signals. Cell.

[CR18] Butler SJ, Bronner ME (2015). From classical to current: analyzing peripheral nervous system and spinal cord lineage and fate. Dev Biol.

[CR19] Cackowski FC, Xu L, Hu B, Cheng SY (2004). Identification of two novel alternatively spliced Neuropilin-1 isoforms. Genomics.

[CR20] Cadilhac C, Bachy I, Forget A, Hodson DJ, Jahannault-Talignani C, Furley AJ, Ayrault O, Mollard P, Sotelo C, Ango F (2021). Excitatory granule neuron precursors orchestrate laminar localization and differentiation of cerebellar inhibitory interneuron subtypes. Cell Rep.

[CR21] Cameron DB, Kasai K, Jiang Y, Hu T, Saeki Y, Komuro H (2009). Four distinct phases of basket/stellate cell migration after entering their final destination (the molecular layer) in the developing cerebellum. Dev Biol.

[CR22] Carter RA, Bihannic L, Rosencrance C, Hadley JL, Tong Y, Phoenix TN, Natarajan S, Easton J, Northcott PA, Gawad C (2018). A single-cell transcriptional atlas of the developing murine cerebellum. Curr Biol.

[CR23] Caviness VS, Rakic P (1978). Mechanisms of cortical development: a view from mutations in mice. Annu Rev Neurosci.

[CR24] Cerrato V, Parmigiani E, Figueres-Onate M, Betizeau M, Aprato J, Nanavaty I, Berchialla P, Luzzati F, de'Sperati C, Lopez-Mascaraque L, Buffo A (2018). Multiple origins and modularity in the spatiotemporal emergence of cerebellar astrocyte heterogeneity. PLoS Biol.

[CR25] Chang JC, Meredith DM, Mayer PR, Borromeo MD, Lai HC, Ou YH, Johnson JE (2013). Prdm13 mediates the balance of inhibitory and excitatory neurons in somatosensory circuits. Dev Cell.

[CR26] Chatterton JE, Awobuluyi M, Premkumar LS, Takahashi H, Talantova M, Shin Y, Cui J, Tu S, Sevarino KA, Nakanishi N, Tong G, Lipton SA, Zhang D (2002). Excitatory glycine receptors containing the NR3 family of NMDA receptor subunits. Nature.

[CR27] Chattopadhyaya B, Di CG, Higashiyama H, Knott GW, Kuhlman SJ, Welker E, Huang ZJ (2004). Experience and activity-dependent maturation of perisomatic GABAergic innervation in primary visual cortex during a postnatal critical period. J Neurosci.

[CR28] Chavas J, Marty A (2003). Coexistence of excitatory and inhibitory GABA synapses in the cerebellar interneuron network. J Neurosci.

[CR29] Chen X, Du Y, Broussard GJ, Kislin M, Yuede CM, Zhang S, Dietmann S, Gabel H, Zhao G, Wang SSH (2022). Transcriptomic mapping uncovers Purkinje neuron plasticity driving learning. Nature.

[CR30] Chizhikov VV, Manto M, Gruol DL, Schmahmann JD, Koibuchi N, Sillitoe RV (2021). Roof plate in cerebellar neurogenesis. Handbook of the cerebellum and cerebellar disorders.

[CR31] Chizhikov VV, Lindgren AG, Currle DS, Rose MF, Monuki ES, Millen KJ (2006). The roof plate regulates cerebellar cell-type specification and proliferation. Development.

[CR32] Chopra R, Wasserman AH, Pulst SM, De Zeeuw CI, Shakkottai VG (2018). Protein kinase C activity is a protective modifier of Purkinje neuron degeneration in cerebellar ataxia. Hum Mol Genet.

[CR33] Consalez GG, Hawkes R (2013). The compartmental restriction of cerebellar interneurons. Front Neural Circ.

[CR34] Consalez GG, Goldowitz D, Casoni F, Hawkes R (2021). Origins, development, and compartmentation of the granule cells of the cerebellum. Front Neural Circuits.

[CR35] Coolen M (2022). Recessive PRDM13 mutations cause fatal perinatal brainstem dysfunction with cerebellar hypoplasia and disrupt Purkinje cell differentiation. Am J Hum Genet.

[CR36] Crino PB, Eberwine J (1996). Molecular characterization of the dendritic growth cone: regulated mRNA transport and local protein synthesis. Neuron.

[CR37] de Luca A, Parmigiani E, Tosatto G, Martire S, Hoshino M, Buffo A, Leto K, Rossi F (2015). Exogenous sonic hedgehog modulates the pool of GABAergic interneurons during cerebellar development. Cerebellum.

[CR38] Delile J, Rayon T, Melchionda M, Edwards A, Briscoe J, Sagner A (2019). Single cell transcriptomics reveals spatial and temporal dynamics of gene expression in the developing mouse spinal cord. Development.

[CR39] Dhar M, Hantman AW, Nishiyama H (2018). Developmental pattern and structural factors of dendritic survival in cerebellar granule cells in vivo. Sci Rep.

[CR40] Dieudonne S, Dumoulin A (2000). Serotonin-driven long-range inhibitory connections in the cerebellar cortex. J Neurosci.

[CR41] Dos Reis R, Kornobis E, Pereira A, Tores F, Carrasco J, Gautier C, Jahannault-Talignani C, Nitschke P, Muchardt C, Schlosser A, Maric HM, Ango F, Allemand E (2022). Complex regulation of Gephyrin splicing is a determinant of inhibitory postsynaptic diversity. Nat Commun.

[CR42] Dumontier D, Mailhes-Hamon C, Supplisson S, Dieudonne S (2023). Neurotransmitter content heterogeneity within an interneuron class shapes inhibitory transmission at a central synapse. Front Cell Neurosci.

[CR43] Fangma Y, Liu M, Liao J, Chen Z, Zheng Y (2023). Dissecting the brain with spatially resolved multi-omics. J Pharm Anal.

[CR44] Farini D, Cesari E, Weatheritt RJ, La SG, Naro C, Pagliarini V, Bonvissuto D, Medici V, Guerra M, Di PC, Rizzo FR, Musella A, Carola V, Centonze D, Blencowe BJ, Marazziti D, Sette C (2020). A dynamic splicing program ensures proper synaptic connections in the developing cerebellum. Cell Rep.

[CR45] Feng H, Moakley DF, Chen S, McKenzie MG, Menon V, Zhang C (2021). Complexity and graded regulation of neuronal cell-type-specific alternative splicing revealed by single-cell RNA sequencing. Proc Natl Acad Sci USA.

[CR46] Fleming JT, He W, Hao C, Ketova T, Pan FC, Wright CC, Litingtung Y, Chiang C (2013). The purkinje neuron acts as a central regulator of spatially and functionally distinct cerebellar precursors. Dev Cell.

[CR47] Gavalas A, Davenne M, Lumsden A, Chambon P, RiJli FM (1997). Role of Hoxa-2 in axon pathfinding and rostral hindbrain patterning. Development.

[CR48] Geurts FJ, De Schutter E, Dieudonne S (2003). Unraveling the cerebellar cortex: cytology and cellular physiology of large-sized interneurons in the granular layer. Cerebellum.

[CR49] Gliem M, Weisheit G, Mertz KD, Endl E, Oberdick J, Schilling K (2006). Expression of classical cadherins in the cerebellar anlage: quantitative and functional aspects. Mol Cell Neurosci.

[CR50] Gomez-Gonzalez GB, Martinez-Torres A (2021). Inter-fastigial projections along the roof of the fourth ventricle. Brain Struct Funct.

[CR51] Grimaldi P, Parras C, Guillemot F, Rossi F, Wassef M (2009). Origins and control of the differentiation of inhibitory interneurons and glia in the cerebellum. Dev Biol.

[CR52] Groteklaes A, Bönisch C, Eiberger B, Christ A, Schilling K (2020). Developmental maturation of the cerebellar white matter—an instructive environment for cerebellar inhibitory interneurons. Cerebellum.

[CR53] Haldipur P (2019). Spatiotemporal expansion of primary progenitor zones in the developing human cerebellum. Science.

[CR54] Haldipur P, Millen KJ, Aldinger KA (2022). Human cerebellar development and transcriptomics: implications for neurodevelopmental disorders. Annu RevNeurosci.

[CR55] Halverson HE, Kim J, Khilkevich A, Mauk MD, Augustine GJ (2022). Feedback inhibition underlies new computational functions of cerebellar interneurons. Elife.

[CR56] Hamre KM, Goldowitz D (1996). Analysis of gene action in the meander tail mouse: examination of cerebellar phenotype and mitotic activity of granule cell neuroblasts. J Comp Neurol.

[CR57] Hanotel J, Bessodes N, Thelie A, Hedderich M, Parain K, Van Driessche B, Brando KO, Kricha S, Jorgensen MC, Grapin-Botton A, Serup P, Van LC, Perron M, Pieler T, Henningfeld KA, Bellefroid EJ (2014). The Prdm13 histone methyltransferase encoding gene is a Ptf1a-Rbpj downstream target that suppresses glutamatergic and promotes GABAergic neuronal fate in the dorsal neural tube. Dev Biol.

[CR58] Hao Y (2021). Integrated analysis of multimodal single-cell data. Cell.

[CR59] Hargrave M, Karunaratne A, Cox L, Wood S, Koopman P, Yamada T (2000). The HMG box transcription factor gene Sox14 marks a novel subset of ventral interneurons and is regulated by sonic hedgehog. Dev Biol.

[CR60] Hashimoto R, Hori K, Owa T, Miyashita S, Dewa K, Masuyama N, Sakai K, Hayase Y, Seto Y, Inoue YU, Inoue T, Ichinohe N, Kawaguchi Y, Akiyama H, Koizumi S, Hoshino M (2016). Origins of oligodendrocytes in the cerebellum, whose development is controlled by the transcription factor, Sox9. Mech Dev.

[CR61] Hauser D, Behr K, Konno K, Schreiner D, Schmidt A, Watanabe M, Bischofberger J, Scheiffele P (2022). Targeted proteoform mapping uncovers specific Neurexin-3 variants required for dendritic inhibition. Neuron.

[CR62] Hecker D, Kappler J, Glassmann A, Schilling K, Alt W (2008). Image analysis of time-lapse movies-A precision control guided approach to correct motion artefacts. J Neurosci Methods.

[CR63] Heckroth JA (1994). Quantitative morphological analysis of the cerebellar nuclei in normal and lurcher mutant mice. I. Morphology and cell number. J Comp Neurol.

[CR64] Hendricks C, Dubail J, Brohee L, Delforge Y, Colige A, Deroanne C (2016). A novel physiological glycosaminoglycan-deficient splice variant of neuropilin-1 is anti-tumorigenic in vitro and in vivo. PLoS One.

[CR65] Hendrikse LD (2022). Failure of human rhombic lip differentiation underlies medulloblastoma formation. Nature.

[CR66] Henke RM, Savage TK, Meredith DM, Glasgow SM, Hori K, Dumas J, MacDonald RJ, Johnson JE (2009). Neurog2 is a direct downstream target of the Ptf1a-Rbpj transcription complex in dorsal spinal cord. Development.

[CR67] Hibi M, Shimizu T (2012). Development of the cerebellum and cerebellar neural circuits. Dev Neurobiol.

[CR68] Hibi M, Matsuda K, Takeuchi M, Shimizu T, Murakami Y (2017). Evolutionary mechanisms that generate morphology and neural-circuit diversity of the cerebellum. Dev Growth Differ.

[CR69] Hidalgo-Sanchez M, Andreu-Cervera A, Villa-Carballar S, Echevarria D (2022). An update on the molecular mechanism of the vertebrate isthmic organizer development in the context of the neuromeric model. Front Neuroanat.

[CR70] Holt CE, Schuman EM (2013). The central dogma decentralized: new perspectives on RNA function and local translation in neurons. Neuron.

[CR71] Hong K, Nishiyama M, Henley J, Tessier-Lavigne M, Poo MM (2000). Calcium signalling in the guidance of nerve growth by netrin-1. Nature.

[CR72] Hörnblad A, Bastide S, Langenfeld K, Langa F, Spitz F (2021). Dissection of the Fgf8 regulatory landscape by in vivo CRISPR-editing reveals extensive intra- and inter-enhancer redundancy. Nat Commun.

[CR73] Hoshino M, Nakamura S, Mori K, Kawauchi T, Terao M, Nishimura YV, Fukuda A, Fuse T, Matsuo N, Sone M, Watanabe M, Bito H, Terashima T, Wright CV, Kawaguchi Y, Nakao K, Nabeshima Y (2005). Ptf1a, a bHLH transcriptional gene, defines GABAergic neuronal fates in cerebellum. Neuron.

[CR74] Huang X, Liu J, Ketova T, Fleming JT, Grover VK, Cooper MK, Litingtung Y, Chiang C (2010). Transventricular delivery of Sonic hedgehog is essential to cerebellar ventricular zone development. Proc Natl Acad Sci USA.

[CR75] Huang C, Gammon SJ, Dieterle M, Huang RH, Likins L, Ricklefs RE (2014). Dramatic increases in number of cerebellar granule-cell-Purkinje-cell synapses across several mammals. Mamm Biol.

[CR76] Huang X, Ye Q, Chen M, Li A, Mi W, Fang Y, Zaytseva YY, O'Connor KL, Vander Kooi CW, Liu S, She QB (2019). N-glycosylation-defective splice variants of neuropilin-1 promote metastasis by activating endosomal signals. Nat Commun.

[CR77] Huard JMT, Forster CC, Carter ML, Sicinski P, Ross ME (1999). Cerebellar histogenesis is disturbed in mice lacking cyclin D2. Development.

[CR78] Ibanez CF, Paratcha G, Ledda F (2020). RET-independent signaling by GDNF ligands and GFRalpha receptors. Cell Tissue Res.

[CR79] Ito M (1984). The cerebellum and neural control.

[CR80] Jean-Xavier C, Mentis GZ, O'Donovan MJ, Cattaert D, Vinay L (2007). Dual personality of GABA/glycine-mediated depolarizations in immature spinal cord. Proc Natl Acad Sci USA.

[CR81] Jensen P, Zoghbi HY, Goldowitz D (2002). Dissection of the cellular and molecular events that position cerebellar Purkinje cells: a study of the math1 null-mutant mouse. J Neurosci.

[CR82] Jiang D, Zhang Y, Hart RP, Chen J, Herrup K, Li J (2015). Alteration in 5-hydroxymethylcytosine-mediated epigenetic regulation leads to Purkinje cell vulnerability in ATM deficiency. Brain.

[CR83] Joglekar A (2021). A spatially resolved brain region- and cell type-specific isoform atlas of the postnatal mouse brain. Nat Commun.

[CR84] Ju J, Liu Q, Zhang Y, Liu Y, Jiang M, Zhang L, He X, Peng C, Zheng T, Lu QR, Li H (2016). Olig2 regulates Purkinje cell generation in the early developing mouse cerebellum. Sci Rep.

[CR85] Kano M, Watanabe T, Uesaka N, Watanabe M (2018). Multiple phases of climbing fiber synapse elimination in the developing cerebellum. Cerebellum.

[CR86] Katsuyama T, Kadoya M, Shirai M, Sasai N (2022). Sox14 is essential for initiation of neuronal differentiation in the chick spinal cord. Dev Dyn.

[CR87] Kebschull JM, Casoni F, Consalez GG, Goldowitz D, Hawkes R, Ruigrok TJH, Schilling K, Wingate R, Wu J, Yeung J, Uusisaari MY (2023). Cerebellum lecture: the cerebellar nuclei-core of the cerebellum. Cerebellum.

[CR88] Kebschull JM, Richman EB, Ringach N, Friedmann D, Albarran E, Kolluru SS, Jones RC, Allen WE, Wang Y, Cho SW, Zhou H, Ding JB, Chang HY, Deisseroth K, Quake SR, Luo L (2020) Cerebellar nuclei evolved by repeatedly duplicating a conserved cell-type set. Science 370:eabd5059. https://github.com/justuskebschull/CNcode_final 10.1126/science.abd5059 PMC8510508 33335034

[CR89] Khouri-Farah N, Guo Q, Morgan K, Shin J, Li JY (2022). Integrated single-cell transcriptomic and epigenetic analyses of cell-state transition and lineage commitment in the embryonic mouse cerebellum. Sci Adv.

[CR90] Kim J, Augustine GJ (2021). Molecular layer interneurons: key elements of cerebellar network computation and behavior. Neuroscience.

[CR91] Kim EJ, Battiste J, Nakagawa Y, Johnson JE (2008). Ascl1 (Mash1) lineage cells contribute to discrete cell populations in CNS architecture. Mol Cell Neurosci.

[CR92] Komine O, Nagaoka M, Hiraoka Y, Hoshino M, Kawaguchi Y, Pear WS, Tanaka K (2011). RBP-J promotes the maturation of neuronal progenitors. Dev Biol.

[CR93] Korbo L, Andersen BB, Ladefoged O, Moler A (1993). Total numbers of various cell types in rat cerebellar cortex estimated using an unbiased stereological method. Brain Res.

[CR94] Kozareva V, Martin C, Osorno T, Rudolph S, Guo C, Vanderburg C, Nadaf N, Regev A, Regehr WG, Macosko E (2021). A transcriptomic atlas of mouse cerebellar cortex comprehensively defines cell types. Nature.

[CR95] Kwon D (2023). The quest to map the mouse brain. Nature.

[CR96] Laine J, Axelrad H (1994). The candelabrum cell: a new interneuron in the cerebellar cortex. J Comp Neurol.

[CR97] Laine J, Axelrad H (1996). Morphology of the Golgi-impregnated Lugaro cell in the rat cerebellar cortex: a reappraisal with a description of its axon. J Comp Neurol.

[CR98] Laine J, Axelrad H (2002). Extending the cerebellar Lugaro cell class. Neuroscience.

[CR99] Lange W (1974). Regional differences in the distribution of golgi cells in the cerebellar cortex of man and some other mammals. Cell Tissue Res.

[CR100] Lange W (1975). Cell number and cell density in the cerebellar cortex of man and some other mammals. Cell Tissue Res.

[CR101] Lein ES et al (2007) Genome-wide atlas of gene expression in the adult mouse brain. Nature 445:168–176. https://developingmouse.brain-map.org/10.1038/nature05453 17151600

[CR102] Leto K, Carletti B, Williams IM, Magrassi L, Rossi F (2006). Different types of cerebellar GABAergic interneurons originate from a common pool of multipotent progenitor cells. J Neurosci.

[CR103] Leto K, Bartolini A, Yanagawa Y, Obata K, Magrassi L, Schilling K, Rossi F (2009). Laminar fate and phenotype specification of cerebellar GABAergic interneurons. J Neurosci.

[CR104] Leto K, Bartolini A, Rossi F (2010). The prospective white matter: an atypical neurogenic niche in the developing cerebellum. Arch Ital Biol.

[CR105] Leto K, Rolando C, Rossi F (2012). The genesis of cerebellar GABAergic neurons: fate potential and specification mechanisms. Front Neuroanat.

[CR106] Leto K (2016). Consensus paper: cerebellar development. Cerebellum.

[CR107] Li W, Chen L, Fleming JT, Brignola E, Zavalin K, Lagrange A, Rex T, Heiney SA, Wojaczynski GJ, Medina JF, Chiang C (2022). Dendritic inhibition by shh signaling-dependent stellate cell pool is critical for motor learning. J Neurosci.

[CR108] Liebl DJ, Morris CJ, Henkemeyer M, Parada LF (2003). mRNA expression of ephrins and Eph receptor tyrosine kinases in the neonatal and adult mouse central nervous system. J Neurosci Res.

[CR109] Lin AC, Holt CE (2007). Local translation and directional steering in axons. EMBO J.

[CR110] Lin PY, Chen LY, Zhou P, Lee SJ, Trotter JH, Südhof TC (2023). Neurexin-2 restricts synapse numbers and restrains the presynaptic release probability by an alternative splicing-dependent mechanism. Proc Natl Acad Sci USA.

[CR111] Lind D, Franken S, Kappler J, Jankowski J, Schilling K (2005). Characterization of the neuronal marker NeuN as a multiply phosphorylated antigen with discrete subcellular localization. J Neurosci Res.

[CR112] Lisabeth EM, Falivelli G, Pasquale EB (2013). Eph receptor signaling and ephrins. Cold Spring Harb Perspect Biol.

[CR113] Liu B, Liu Z, Chen T, Li H, Qiang B, Yuan J, Peng X, Qiu M (2007). Selective expression of Bhlhb5 in subsets of early-born interneurons and late-born association neurons in the spinal cord. Dev Dyn.

[CR114] Lowenstein ED, Rusanova A, Stelzer J, Hernaiz-Llorens M, Schroer AE, Epifanova E, Bladt F, Isik EG, Buchert S, Jia S, Tarabykin V, Hernandez-Miranda LR (2021). Olig3 regulates early cerebellar development. Elife.

[CR115] Luo Z (2022). Human fetal cerebellar cell atlas informs medulloblastoma origin and oncogenesis. Nature.

[CR116] Ma TC, Vong KI, Kwan KM (2020). Spatiotemporal decline of BMP signaling activity in neural progenitors mediates fate transition and safeguards neurogenesis. Cell Rep.

[CR117] Manto MU, Gruol DL, Schmahmann JD, Koibuchi N, Sillitoe RV (2021). Handbook of the cerebellum and cerebellar disorders.

[CR118] Maricich SM, Herrup K (1999). Pax-2 expression defines a subset of GABAergic interneurons and their precursors in the developing murine cerebellum. J Neurobiol.

[CR119] Marsh S (2023) scCustomize: custom visualizations & functions for streamlined analyses of single cell sequencing. https://github.com/samuel-marsh/scCustomize

[CR120] Maxeiner S, Glassmann A, Kao HT, Schilling K (2013). The molecular basis of the specificity and cross-reactivity of the NeuN epitope of the neuron-specific splicing regulator, Rbfox3. Histochem Cell Biol.

[CR121] Mazin PV, Khaitovich P, Cardoso-Moreira M, Kaessmann H (2021) Alternative splicing during mammalian organ development. Nat Genet 53:925–934. https://apps.kaessmannlab.org/alternative-splicing/10.1038/s41588-021-00851-w PMC8187152 33941934

[CR122] Mendonsa S, von Kügelgen N, Dantsuji S, Ron M, Breimann L, Baranovskii A, Lödige I, Kirchner M, Fischer M, Zerna N, Bujanic L, Mertins P, Ulitsky I, Chekulaeva M (2023). Massively parallel identification of mRNA localization elements in primary cortical neurons. Nat Neurosci.

[CR123] Ming G, Song H, Berninger B, Holt CE, Tessier-Lavigne M, Poo MM (1997). cAMP-dependent growth cone guidance by Netrin-1. Neuron.

[CR124] Miyazaki T, Yamasaki M, Tanaka KF, Watanabe M (2021). Compartmentalized input-output organization of Lugaro cells in the cerebellar cortex. Neuroscience.

[CR125] Mona B, Uruena A, Kollipara RK, Ma Z, Borromeo MD, Chang JC, Johnson JE (2017). Repression by PRDM13 is critical for generating precision in neuronal identity. Elife.

[CR126] Napieralski JA, Eisenman LM (1993). Developmental analysis of the external granule cell layer in the meander tail mutant mouse: do cerebellar microneurons have independent progenitors?. Dev Dyn.

[CR127] Napieralski JA, Eisenman LM (1996). Further evidence for a unique developmental compartment in the cerebellum of the meander tail mutant mouse as revealed by the quantitative analysis of Purkinje cells. J Comp Neurol.

[CR128] Nawy S (1999). The metabortropic receptor mGluR6 may signal through G_o_, but not phosphodiesterase, in retinal bipolar cells. J Neurosci.

[CR129] Nelson BR, Hartman BH, Ray CA, Hayashi T, Bermingham-McDonogh O, Reh TA (2009). Acheate-scute like 1 (Ascl1) is required for normal delta-like (Dll) gene expression and notch signaling during retinal development. Dev Dyn.

[CR130] Neubüser A, Peters H, Balling R, Martin GR (1997). Antagonistic interactions between FGF and BMP signaling pathways: a mechanism for positioning the sites of tooth formation. Cell.

[CR131] Nguyen TM, Schreiner D, Xiao L, Traunmüller L, Bornmann C, Scheiffele P (2016). An alternative splicing switch shapes neurexin repertoires in principal neurons versus interneurons in the mouse hippocampus. Elife.

[CR132] Oberdick J, Levinthal F, Levinthal C (1988). A Purkinje cell differentiation marker shows a partial DNA sequence homology to the cellular sis/PDGF2 gene. Neuron.

[CR133] Oberdick J, Smeyne RJ, Mann JR, Zackson S, Morgan JI (1990). A promoter that drives transgene expression in cerebellar Purkinje and retinal bipolar neurons. Science.

[CR134] Ohtoshi A, Behringer RR (2004). Neonatal lethality, dwarfism, and abnormal brain development in Dmbx1 mutant mice. Mol Cell Biol.

[CR135] Ohtoshi A, Nishijima I, Justice MJ, Behringer RR (2002). Dmbx1, a novel evolutionarily conserved paired-like homeobox gene expressed in the brain of mouse embryos. Mech Dev.

[CR136] Osorno T, Rudolph S, Nguyen T, Kozareva V, Nadaf NM, Norton A, Macosko EZ, Lee WCA, Regehr WG (2022). Candelabrum cells are ubiquitous cerebellar cortex interneurons with specialized circuit properties. Nature Neurosci.

[CR137] Palay SL, Chan-Palay V (1974). Cerebellar Cortex. Cytology and organization.

[CR138] Pan Q, Shai O, Lee LJ, Frey BJ, Blencowe BJ (2008). Deep surveying of alternative splicing complexity in the human transcriptome by high-throughput sequencing. Nat Genet.

[CR139] Paratcha G, Ledda F, Ibanez CF (2003). The neural cell adhesion molecule NCAM is an alternative signaling receptor for GDNF family ligands. Cell.

[CR140] Park H, Kim T, Kim J, Yamamoto Y, Tanaka-Yamamoto K (2019). Inputs from sequentially developed parallel fibers are required for cerebellar organization. Cell Rep.

[CR141] Pascual M, Abasolo I, Mingorance-Le Meur A, Martinez A, Del Rio JA, Wright CV, Real FX, Soriano E (2007). Cerebellar GABAergic progenitors adopt an external granule cell-like phenotype in the absence of Ptf1a transcription factor expression. Proc Natl Acad Sci USA.

[CR142] Perez JD, Fusco CM, Schuman EM (2021). A Functional dissection of the mRNA and locally synthesized protein population in neuronal dendrites and axons. Annu Rev Genet.

[CR143] Pfeffer PL, Payer B, Reim G, di Magliano MP, Busslinger M (2002). The activation and maintenance of Pax2 expression at the mid-hindbrain boundary is controlled by separate enhancers. Development.

[CR144] Pierani A, Moran-Rivard L, Sunshine MJ, Littman DR, Goulding M, Jessell TM (2001). Control of interneuron fate in the developing spinal cord by the progenitor homeodomain protein Dbx1. Neuron.

[CR145] Pilotto F, Douthwaite C, Diab R, Ye X, Al QZ, Tietje C, Mounassir M, Odriozola A, Thapa A, Buijsen RAM, Lagache S, Uldry AC, Heller M, Müller S, van Roon-Mom WMC, Zuber B, Liebscher S, Saxena S (2023). Early molecular layer interneuron hyperactivity triggers Purkinje neuron degeneration in SCA1. Neuron.

[CR146] Prekop HT, Kroiss A, Rook V, Zagoraiou L, Jessell TM, Fernandes C, Delogu A, Wingate RJT (2018). Sox14 is required for a specific subset of cerebello-olivary projections. J Neurosci.

[CR147] Prestori F, Mapelli L, D'Angelo E (2019). Diverse neuron properties and complex network dynamics in the cerebellar cortical inhibitory circuit. Front Mol Neurosci.

[CR148] R Core Team (2023). R: A language and environment for statistical computing.

[CR149] Ramirez M, Badayeva Y, Yeung J, Wu J, Abdalla-Wyse A, Yang E, Trost B, Scherer SW, Goldowitz D (2022). Temporal analysis of enhancers during mouse cerebellar development reveals dynamic and novel regulatory functions. Elife.

[CR150] Ramon y Cajal S (1909). Histologie du système nerveux de l'homme et des vertébrés II.

[CR151] Ross SE, McCord AE, Jung C, Atan D, Mok SI, Hemberg M, Kim TK, Salogiannis J, Hu L, Cohen S (2012). Bhlhb5 and Prdm8 form a repressor complex involved in neuronal circuit assembly. Neuron.

[CR152] Sagner A, Briscoe J (2019). Establishing neuronal diversity in the spinal cord: a time and a place. Development.

[CR153] Sato T, Joyner AL (2009). The duration of Fgf8 isthmic organizer expression is key to patterning different tectal-isthmo-cerebellum structures. Development.

[CR154] Schilling K, Manto M, Gruol DL, Schmahmann JD, Koibuchi N, Sillitoe RV (2019). Specification and development of GABAergic interneurons. Handbook of the cerebellum and cerebellar disorders.

[CR155] Schilling K, Oberdick J (2010). The treasury of the commons: making use of public gene expression resources to better characterize the molecular diversity of inhibitory interneurons in the cerebellar cortex. Cerebellum.

[CR156] Schilling K, Dickinson MH, Connor JA, Morgan JI (1991). Electrical activity in cerebellar cultures determines Purkinje cell dendritic growth patterns. Neuron.

[CR157] Schilling K, Luk D, Curran T, Morgan JI (1991). Regulation of a fos-lacZ fusion gene: a paradigm for quantitative analysis of stimulus-transcription coupling. Proc Natl Acad Sci USA.

[CR158] Schilling K, Oberdick J, Rossi F, Baader SL (2008). Besides Purkinje cells and granule neurons: an appraisal of the cell biology of the interneurons of the cerebellar cortex. Histochem Cell Biol.

[CR159] Sergaki MC, Lopez-Ramos JC, Stagkourakis S, Gruart A, Broberger C, Delgado-Garcia JM, Ibanez CF (2017). Compromised survival of cerebellar molecular layer interneurons lacking GDNF receptors GFRα1 or RET impairs normal cerebellar motor learning. Cell Rep.

[CR160] Seto Y, Ishiwata S, Hoshino M (2014). Characterization of Olig2 expression during cerebellar development. Gene Expr Patterns.

[CR161] Seto Y (2014). Temporal identity transition from Purkinje cell progenitors to GABAergic interneuron progenitors in the cerebellum. Nat Commun.

[CR162] Shi M, Hu ZL, Zheng MH, Song NN, Huang Y, Zhao G, Han H, Ding YQ (2012). Notch-Rbpj signaling is required for the development of noradrenergic neurons in the mouse locus coeruleus. J Cell Sci.

[CR163] Shi H, He Y, Zhou Y, Huang J, Maher K, Wang B, Tang Z, Luo S, Tan P, Wu M, Lin Z, Ren J, Thapa Y, Tang X, Chan KY, Deverman BE, Shen H, Liu A, Liu J, Wang X (2023). Spatial atlas of the mouse central nervous system at molecular resolution. Nature.

[CR164] Siebel C, Lendahl U (2017). Notch signaling in development, tissue homeostasis, and disease. Physiol Rev.

[CR165] Simat M, Parpan F, Fritschy JM (2007). Heterogeneity of glycinergic and gabaergic interneurons in the granule cell layer of mouse cerebellum. J Comp Neurol.

[CR166] Simeone A, D'Apice MR, Nigro V, Casanova J, Graziani F, Acampora D, Avantaggiato V (1994). Orthopedia, a novel homeobox-containing gene expressed in the developing CNS of both mouse and Drosophila. Neuron.

[CR167] Skaggs K, Martin DM, Novitch BG (2011). Regulation of spinal interneuron development by the Olig-related protein Bhlhb5 and Notch signaling. Development.

[CR168] Smeyne RJ, Schilling K, Robertson LM, Luk D, Oberdick J, Curran T, Morgan JI (1992). Fos-lacZ transgenic mice: mapping sites of gene induction in the central nervous system. Neuron.

[CR169] Smeyne RJ, Chu T, Lewin A, Bian F, Crisman F, Kunsch C, Lira SA, Oberdick J (1995). Local control of granule cell generation by cerebellar Purkinje cells. Mol Cell Neurosci.

[CR170] Smirnow AE (1897). Ueber eine besondere Art von Nervenzellen der Molecular Schicht des Kleinhirns bei erwachsenen Säugetieren und beim Menschen. AnatAnz.

[CR171] Spitzer NC (2015). Neurotransmitter switching? No surprise. Neuron.

[CR172] Sudarov A, Turnbull RK, Kim EJ, Lebel-Potter M, Guillemot F, Joyner AL (2011). Ascl1 genetics reveals insights into cerebellum local circuit assembly. J Neurosci.

[CR173] Sultan F, Bower JM (1998). Quantitative Golgi study of the rat cerebellar molecular layer interneurons using principal component analysis. J Comp Neurol.

[CR174] Surchev L, Nazwar TA, Weisheit G, Schilling K (2007). Developmental increase of total cell numbers in the murine cerebellum. Cerebellum.

[CR175] Takahashi T, Holland PW (2004). Amphioxus and ascidian Dmbx homeobox genes give clues to the vertebrate origins of midbrain development. Development.

[CR176] Tantra M, Guo L, Kim J, Zainolabidin N, Eulenburg V, Augustine GJ, Chen AI (2018). Conditional deletion of Cadherin 13 perturbs Golgi cells and disrupts social and cognitive behaviors. Genes Brain Behav.

[CR177] Telley L, Cadilhac C, Cioni JM, Saywell V, Jahannault-Talignani C, Huettl RE, Sarrailh-Faivre C, Dayer A, Huber AB, Ango F (2016). Dual function of NRP1 in axon guidance and subcellular target recognition in cerebellum. Neuron.

[CR178] Tzatzalos E, Smith SM, Doh ST, Hao H, Li Y, Wu A, Grumet M, Cai L (2012). A cis-element in the Notch1 locus is involved in the regulation of gene expression in interneuron progenitors. Dev Biol.

[CR179] Vladoiu MC (2019). Childhood cerebellar tumours mirror conserved fetal transcriptional programs. Nature.

[CR180] Wall NA, Hogan BL (1995). Expression of bone morphogenetic protein-4 (BMP-4), bone morphogenetic protein-7 (BMP-7), fibroblast growth factor-8 (FGF-8) and sonic hedgehog (SHH) during branchial arch development in the chick. Mech Dev.

[CR181] Wang WX, Lefebvre JL (2022). Morphological pseudotime ordering and fate mapping reveal diversification of cerebellar inhibitory interneurons. Nat Commun.

[CR182] Wang ET, Sandberg R, Luo S, Khrebtukova I, Zhang L, Mayr C, Kingsmore SF, Schroth GP, Burge CB (2008). Alternative isoform regulation in human tissue transcriptomes. Nature.

[CR183] Wanner I, Baader SL, Brich M, Oberdick J, Schilling K (1997). Subcellular localization of specific mRNAs and their protein products in Purkinje cells by combined fluorescent in situ hybridization and immunocytochemistry. Histochem Cell Biol.

[CR184] Wassef M, Manto M, Gruol DL, Schmahmann JD, Koibuchi N, Rossi F (2022). Specification of the cerebellar territory. Handbook of the cerebellum and cerebellar disorders.

[CR185] Wefers AK, Haberlandt C, Tekin NB, Fedorov DA, Timmermann A, van der Want JJL, Chaudhry FA, Steinhauser C, Schilling K, Jabs R (2017). Synaptic input as a directional cue for migrating interneuron precursors. Development.

[CR186] Weisheit G, Gliem M, Endl E, Pfeffer PL, Busslinger M, Schilling K (2006). Postnatal development of the murine cerebellar cortex: formation and early dispersal of basket, stellate and Golgi neurons. Eur J Neurosci.

[CR187] Wen W, Lin CY, Niu L (2017). R/G editing in GluA2R(flop) modulates the functional difference between GluA1 flip and flop variants in GluA1/2R heteromeric channels. Sci Rep.

[CR188] Wen WX, Mead AJ, Thongjuea S (2023). MARVEL: an integrated alternative splicing analysis platform for single-cell RNA sequencing data. Nucleic Acids Res.

[CR189] Weyer A, Schilling K (2003). Developmental and cell type-specific expression of the neuronal marker NeuN in the murine cerebellum. J Neurosci Res.

[CR190] Whittaker DE (2021). A recessive PRDM13 mutation results in congenital hypogonadotropic hypogonadism and cerebellar hypoplasia. J Clin Invest.

[CR191] Williamson D, Schwalbe EC, Hicks D, Aldinger KA, Lindsey JC, Crosier S, Richardson S, Goddard J, Hill RM, Castle J, Grabovska Y, Hacking J, Pizer B, Wharton SB, Jacques TS, Joshi A, Bailey S, Clifford SC (2022). Medulloblastoma group 3 and 4 tumors comprise a clinically and biologically significant expression continuum reflecting human cerebellar development. Cell Rep.

[CR192] Wilson L, Maden M (2005). The mechanisms of dorsoventral patterning in the vertebrate neural tube. Dev Biol.

[CR193] Wizeman JW, Guo Q, Wilion EM, Li JY (2019). Specification of diverse cell types during early neurogenesis of the mouse cerebellum. Elife.

[CR194] Wong L, Power N, Miles A, Tropepe V (2015). Mutual antagonism of the paired-type homeobox genes, vsx2 and dmbx1, regulates retinal progenitor cell cycle exit upstream of ccnd1 expression. Dev Biol.

[CR195] Xu J, Xu M, Wang Y, Mathena RP, Wen J, Zhang P, Furmanski O, Mintz CD (2019). Anesthetics disrupt growth cone guidance cue sensing through actions on the GABAα2 receptor mediated by the immature chloride gradient. Neurotoxicol Teratol.

[CR196] Zhang R, Zhang X, Bian F, Pu XA, Schilling K, Oberdick J (2008). 3'UTR-dependent localization of a Purkinje cell messenger RNA in dendrites. Cerebellum.

[CR197] Zhang T, Liu T, Mora N, Guegan J, Bertrand M, Contreras X, Hansen AH, Streicher C, Anderle M, Danda N, Tiberi L, Hippenmeyer S, Hassan BA (2021). Generation of excitatory and inhibitory neurons from common progenitors via Notch signaling in the cerebellum. Cell Rep.

[CR198] Zheng P, Zhang N, Ren D, Yu C, Zhao B, Zhang Y (2023). Integrated spatial transcriptome and metabolism study reveals metabolic heterogeneity in human injured brain. Cell Rep Med.

[CR199] Zordan P, Croci L, Hawkes R, Consalez GG (2008). Comparative analysis of proneural gene expression in the embryonic cerebellum. Dev Dyn.

